# Evidence for a Role of CCR6+ T Cells in Chronic Thromboembolic Pulmonary Hypertension

**DOI:** 10.3389/fimmu.2022.861450

**Published:** 2022-04-27

**Authors:** Denise van Uden, Thomas Koudstaal, Jennifer A. C. van Hulst, Thierry P. P. van den Bosch, Madelief Vink, Ingrid M. Bergen, Karishma A. Lila, Annemien E. van den Bosch, Paul Bresser, Mirjam Kool, Jan H. von der Thüsen, Rudi W. Hendriks, Karin A. Boomars

**Affiliations:** ^1^ Department of Pulmonary Medicine, Erasmus MC, University Medical Center Rotterdam, Rotterdam, Netherlands; ^2^ Department of Pathology, Erasmus MC, University Medical Center Rotterdam, Rotterdam, Netherlands; ^3^ Department of Cardiology, Erasmus MC, University Medical Center Rotterdam, Rotterdam, Netherlands; ^4^ Department of Respiratory Medicine, OLVG, Amsterdam, Netherlands

**Keywords:** chronic thromboembolic pulmonary hypertension, immunology, T cell, cytokine, CCR6, CTLA4

## Abstract

**Introduction:**

Previous studies have shown an increase of T cells and chemokines in vascular lesions of patients with chronic thromboembolic pulmonary hypertension (CTEPH). However, detailed characterization of these T cells is still lacking, nor have treatment effects been evaluated.

**Methods:**

We included 41 treatment-naive CTEPH patients at diagnosis, 22 patients at 1-year follow-up, and 17 healthy controls (HCs). Peripheral blood T cells were characterized by flow cytometry for subset distribution, cytokine expression and activation marker profile. We used multiplex immunofluorescence to identify CCR6^+^ T cells in endarterectomy tissue from 25 patients.

**Results:**

At diagnosis, proportions of CCR6^+^ CD4^+^ T cells were increased in CTEPH patients compared with HCs. Patients displayed a significantly reduced production capacity of several cytokines including TNFα, IFNγ, GM-CSF and IL-4 in CD4^+^ T cells, and TNFα and IFNγ in CD8^+^ T cells. CD4^+^ and CD8^+^ T cells showed increased expression of the immune checkpoint protein CTLA4. Multivariate analysis separated CTEPH patients from HCs, based on CCR6 and CTLA4 expression. At 1-year follow-up, proportions of CCR6^+^CD4^+^ T cells were further increased, IFNγ and IL-17 production capacity of CD4^+^ T cells was restored. In nearly all vascular lesions we found substantial numbers of CCR6^+^ T cells.

**Conclusion:**

The observed increase of CCR6^+^ T cells and modulation of the IFNγ and IL-17 production capacity of circulating CD4^+^ T cells at diagnosis and 1-year follow-up – together with the presence of CCR6^+^ T cells in vascular lesions - support the involvement of the Th17-associated CCR6^+^ T cell subset in CTEPH.

## Introduction

Chronic thromboembolic pulmonary hypertension (CTEPH) is a debilitating disease occurring as a rare complication following acute pulmonary embolism (1). The combination of occlusion of proximal pulmonary arteries by thrombotic material and secondary microvasculopathy of small vessels (<500nm) leads to increased pulmonary vascular resistance (PVR) and progressive right heart failure. Based on the localization and characterization of the thrombotic lesions in the pulmonary vasculature, therapy may consist of pulmonary endarterectomy (PEA) surgery, balloon pulmonary angioplasty (BPA) intervention, and/or pulmonary hypertension (PH)-specific drug therapy. In most cases, CTEPH patients are operable and eligible for PEA intervention, leading to very good long-term survival in expert centers (2–4). By contrast, when CTEPH patients display mainly peripheral localization of the thrombotic lesions or have considerable co-morbidities, they become inoperable. In that case survival remains poor with a mean 5-year survival of 53-69%, even when treated with specific PAH medication (5, 6). Therefore, more insight into the pathogenesis of CTEPH is urgently needed and would fuel the development of new therapeutic strategies.

Thrombotic lesions of CTEPH patients contain various immune cells, particularly myeloid cells including macrophages and neutrophils (7–10). Myeloid cells are attracted towards the lesions by various chemokines that were found to be increased in plasma or thrombotic lesions of CTEPH patients (7, 8, 11, 12). Circulating neutrophils display an activated state and neutrophil extracellular trap formation has been implicated in CTEPH pathogenesis (13, 14).

By contrast, knowledge on the role of adaptive immunity in CTEPH is limited (15–17). T cells expressing the co-inhibitory marker CD200 are increased in the circulation of CTEPH patients. T cells are abundantly present in vascular lesions, as shown by histological analysis and single-cell RNA analysis on endarterectomy tissue (7, 18, 19). CCL2, produced by many cell types, can also regulate migration of T cells and natural killer cells (20). Myeloid cells in thrombotic lesions can interact with T lymphocytes, which in turn may enhance myeloid cell activity through cytokine production. Inflammatory mediators, such as interleukin (IL)-6, IL-8, IL-10 and tumor necrosis factor-α (TNFα), are increased in serum or plasma of CTEPH patients (7, 11, 12, 21–24). This increase might either promote T cell activation or may be a reflection thereof. Moreover, chemokine CXCL3, which is important for T helper 1 (Th1) cells expressing its receptor CXCR3, is increased in serum and lung tissue (25). The importance of these inflammatory mediators is illustrated by our earlier findings that increased plasma levels of CXCL9 and IL-8 correlated with decreased survival in CTEPH patients (22) and point to involvement of the adaptive immune system.

CD4^+^ T cells can be divided into different functional subsets (26). Th1 (CCR6^-^CXCR3^+^CCR4^-^) cells are induced by IL-12 and secrete interferon γ (IFNγ) to defend against intracellular pathogens. Th2 cells (CCR6^-^CXCR3^-^CCR4^+^) produce IL-4, IL-5 and IL-13 and are involved in defense against helminths. Th17 cells (CCR6^+^) are induced by IL-1β, IL-6 and transforming growth factor β (TGFβ) and secrete IL-17. They protect against extracellular pathogens, but are also involved in the pathophysiology of autoimmune diseases (27). In parallel, CCR6^+^CD8^+^ T cells, known as Tc17 cells, are found in patients with different inflammatory diseases (28, 29). They migrate to similar sites as Th17 cells, attracted by CCL20, and produce IL-17, IFNγ and TNFα. The increase of TNFα and IL-6 in serum of CTEPH patients, suggests a role for CCR6^+^ T cells in disease pathogenesis (11, 12, 23, 24).

In this report, we used flow cytometry to investigate subset division, cytokine production and activation status of circulating T cells from a well-defined, treatment-naive cohort of CTEPH patients. We also investigated how treatment affected this T cell profile and we characterized T cells present in pulmonary vascular lesions.

## Methods

### Subjects and Study Design

Our prospective observational cohort of CTEPH patients has been previously reported (22). Forty-one CTEPH patients were diagnosed according to the ERS/ECSC guidelines (**Table 1**) (30). Similar to prior work from our group, exclusion criteria were incomplete diagnostic work-up and therefore no confirmed PH diagnosis, not treatment-naive, age <18 years, or not capable of understanding or signing informed consent (31). Additionally,17 HCs (41% female, mean age 55.3 ± 12.5), were included with the following exclusion criteria: autoimmune disease, active infectious disease, use of immunomodulatory drugs, history of cardiopulmonary disease. At 1-year follow-up patients visited the outpatient clinic and did not have any active infection. The study protocol was approved by the Erasmus MC medical ethical committee (MEC-2011-392). Written informed consent was provided by all patients and controls. The study was performed conform the principles outlined in the declaration of Helsinki.

**Table 1:** Baseline demographic and patient characteristics.

	CTEPH Baseline (n = 41)	CTEPH Follow-up (n = 22)
➢ **Baseline clinical characteristics**		
Gender, female (%)	20 (49%)	12 (55%)
Age, y	62.0 ± 13.3^1^	64.6 ± 11.1
BMI, kg/m2	29.7 ± 6.3	29.4 ± 6.7
NYHA class 3-4, n (%)	19 (46%)	10 (45%)
6MWT, m	378 ± 140	335 ± 135
NT-pro BNP, pmol/L	115 ± 184	136 ± 213
➢ **Baseline right heart catheterization**		
mPAP, mmHg	39.1 ± 12.7	38.7 ± 13.8
mRAP, mmHg	9.4 ± 6.7	8.9 ± 4.8
Capillary wedge pressure, mmHg	12.3 ± 4.3	11.9 ± 5.2
PVR, wood units	4.8 ± 2.8	5.2 ± 2.7
➢ **Thrombotic lesion localization**		
Central vasculature	16 (39%)	8 (36%)
Mid vasculature	15 (37%)	6 (28%)
Peripheral vasculature	10 (24%)	8 (36%)
➢ **Intervention received (at t= 1-year follow-up)**		
PEA		9 (22%)
PEA (technically operable, yet no surgery performed)		7 (17%)
BPA (within 1-year follow-up)		5 (12%)
BPA (after 1-year follow-up)		6 (15%)
No intervention, only PH-medication		14 (34%)
➢ **PH-Medication**		
At baseline, n (%)	0/41 (0%)	
At 1-year follow-up		
No PH-medication^2^		3/22 (14%)
Mono therapy, n (%)		10/22 (45%)
Duo therapy, n (%)		9/22 (41%)
Triple therapy, n (%)		0/22 (0%)
➢ **Immunomodulatory drugs**		
At baseline, n (%)	0/41 (0%)	
At 1-year follow-up, n (%)		0/22 (0%)

^1^ Data given as mean values ± SD, unless otherwise indicated.

^2^ These CTEPH patients were not on PH-medication due to being technically operable for pulmonary endarterectomy.

### Clinical Data Collection, Follow-Up, and Definition of Endpoints

Hemodynamic and clinical data at diagnosis were collected during the inpatient cardiopulmonary screening visit for analysis of PH (22, 31). Data were collected and stored in PAHTool (version 4.3.5947.29411, Inovoltus), an online electronic case report form. Patients were treated according to the ERS/ESC guidelines (30) and prospectively followed-up by half-yearly scheduled visits to the outpatient clinic. All patients were assessed for eligibility for either a PEA or BPA (**Table 1**). The mean follow-up duration was 51.0 months. Survival was defined as all-cause mortality. In our cohort, 7 out of 41 patients died within the follow-up period.

### Flow Cytometry and Histology of Vascular Lesions

Peripheral blood mononuclear cells were stained for intra- and extracellular markers, using flow cytometry procedures essentially as described previously (32), using the antibodies given in **Supplementary Table 1**. Cell fractions were directly stained for chemokine receptors and extracellular markers for 60 minutes at 4°C. After fixation and permeabilization steps, cells were intracellularly stained for FoxP3 and CTLA4. For the measurement of cytokines, PBMCs were incubated for 4 hours at 37°C in RPMI Medium 1640 + GlutaMAX-I (Gibco) supplemented with 5% fetal bovine serum (Gibco), 10 ng/ml phorbol 12-mysristate 13-acetate (Sigma-Aldrich), 250 ng/ml ionomycin (Sigma-Adrich) and Golgistop (BD Bioscience), after which cells were stained as previously described (33). Non-specific labelling was prevented by blocking Fc receptors using human TruStain FcX (Biolegend) and dead cells were excluded with Fixable Viability Dye Live/Dead eF506 (eBioscience). Data was acquired using a FACSymphony A5 flow cytometer (Beckton Dickinson) and analyzed using FlowJo version 10 (Tree Star Inc software).

Hematoxylin and eosin (H&E) staining and multiplex immunofluorescence were performed as described previously (34). For immunohistochemical analysis of human lung tissue, we performed a 5-plex immunofluorescent multiplex by automated IHC using the Ventana Benchmark Discovery ULTRA (Ventana Medical Systems Inc.). Formalin fixed paraffin embedded (FFPE)-tissue of lung vascular lesions of CTEPH patients were handled and used according to the guidelines of the declaration of Helsinki. This study was conducted in accordance with the guidelines of the Biomedical Scientific Societies (Dutch Federa code of conduct 2011) for the use of anonymized residual tissue obtained during regular treatment.

Slides of 4 µm thick FFPE sections were stained for CD3, CD4, CD8, FOXP3 and CCR6 (**Supplementary Table 2**). In brief, following deparaffinization and heat-induced antigen retrieval with CC1 (#950-224, Ventana) for 32 min, anti-CD3 was incubated for 32 min at 37°C followed by omnimap anti-rabbit HRP (#760-4311, Ventana) and detection with R6G (#760-244, Ventana). An antibody denaturation step was performed with CC2 (#950-123, Ventana) at 100°C for 20 min. Secondly, incubation with anti-FOXP3 was performed for 60 min at 37°C, followed by universal HQ kit (#760-275, Ventana) and detection with DCC (#760-240, Ventana) for 8 min. An antibody denaturation step was performed with CC2 at 100°C for 20 min. Thirdly, anti-CD4 was incubated for 32 min at 37°C, followed by omnimap anti-rabbit HRP (#760-4311, Ventana) and detection with Red610 (#760-245, Ventana) for 8 minutes. An antibody denaturation step was performed with CC2 at 100°C for 20 min. Fourthly, sections were incubated with anti-CCR6 for 120 min at 37°C followed by universal HQ kit (#760-275, Ventana) and detection with Cy5 (#760-238, Ventana) for 8 min. An antibody denaturation step was performed with CC2 at 100°C for 20 min. Lastly, incubation with anti-CD8 was performed for 60 min at 37°C followed by omnimap anti-rabbit HRP (#760-4311, Ventana) and detection with FAM (#760-243, Ventana). Finally, slides were washed in phosphate-buffered saline and mounted with Vectashield containing 4’,6-diamidino-2-phenylindole (Vector laboratories, Peterborough, UK). Analysis of the multiplex staining was performed using Qupath the open software platform for bioimage analysis (version 0.3.0). In short, sections were annotated by simple tissue detection after which cells were identified by the default cell detection command using dapi to identify nuclei. Using simple thresholding for all channels all cells were classified and annotated. These measurements were used for further analysis.

### Principal Component Analysis and Statistical Evaluation

Principal component analysis (PCA) was performed using R and RStudio, and the packages FactoMineR and Factoextra (22, 35).

Statistical evaluations of T cell subsets, cytokine production, activation markers and PCA dimension coordinates were by Mann-Whitney U tests. Paired diagnosis and 1-year follow-up data were analyzed using a Wilcoxon matched-pairs signed rank test. Correlation coefficients were calculated using the nonparametric Spearman correlation. All statistical tests were two-sided; p-values <0.05 were considered statistically significant. Statistical analyses were performed using GraphPad Prism v8 (Graph Pad Software).

## Results

### Increased Proportions of Circulating CCR6^+^CD4^+^ T Cells in Treatment-Naive CTEPH Patients

**Figure 1:**
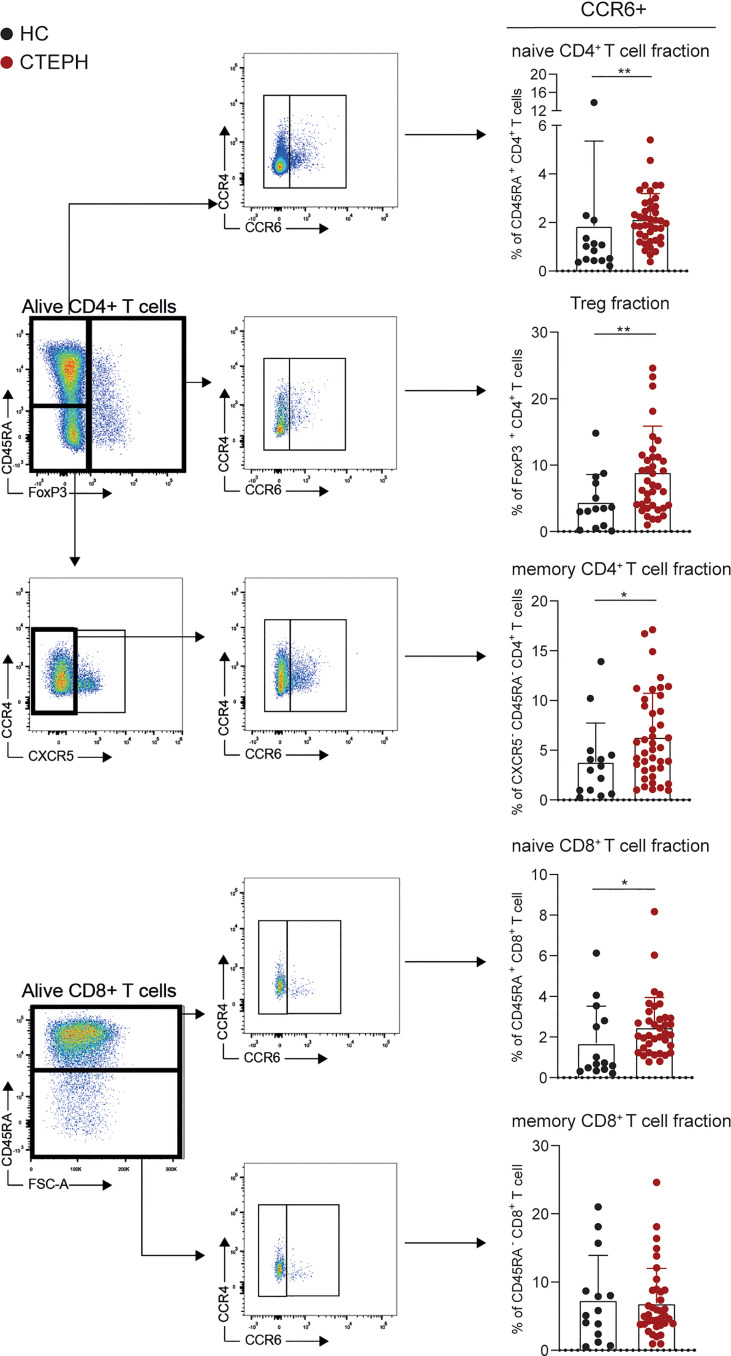
Frequencies of CCR6+ T cells are higher in patients with chronic thromboembolic pulmonary hypertension (CTEPH) than in healthy controls (HCs) at diagnosis. Gating strategy for peripheral blood CCR6^+^ T cell subsets (*left*) and percentages of circulating CCR6^+^ T cells (*right*) of the indicated T cell subsets for HCs and CTEPH patients at diagnosis determined by flow cytometry. Results are presented as mean + standard deviation, Mann-Whitney U test was used for statistical analysis, *p < 0.05, **p < 0.01. Symbols represent values of individual patients or HCs.

We characterized peripheral blood T cells in a cohort of 41 treatment-naive CTEPH patients at diagnosis and 17 HCs (**Table 1**). Five subpopulations were separately analyzed: CD45RA^+^ CD4^+^ T cells (FoxP3^-^; mainly naive T cells, but also CD45RA^+^ TEMRA cells, which are thought to be antigen-experienced T cells re-expressing CD45RA), CD45RA^-^ CD4^+^ T cells (FoxP3^-^CXCR5^-^; memory T cells), FoxP3^+^ CD4^+^ T cells (Tregs and activated T cells), CD45RA^-^ CD8^+^ T cells and CD45RA^+^ CD8^+^ T cells (see **Figure 1** and **Supplementary Figure 1** for gating strategy). The proportions of these five T cell subpopulations did not differ between CTEPH patients and HCs (data not shown).

We used the expression of surface chemokine receptors to distinguish distinct CD45RA^-^ CD4^+^ memory T cell subsets, including Th1 (CCR6^-^CXCR3^+^CCR4^-^), Th2 (CCR6^-^CXCR3^-^CCR4^+^), Th17 (CCR6^+^) and follicular T helper cells (Tfh; CXCR5^+^). Whereas the frequencies of Th1, Th2 and Tfh cells did not differ between CTEPH patients and HCs (data not shown), we observed an increase in Th17 cells within the memory CD4^+^ T cells (**Figure 1**). A sub-analysis of CCR6^+^ CD4^+^ T cells, identifying CXCR3^-^CCR4^+^ Th17, CXCR3^+^CCR4^-^ Th17.1, CXCR3^+^CCR4^+^ double positive (DP) and CXCR3^-^CCR4^-^ double negative (DN) cells, showed a particularly strong increase in the proportions of CXCR3^-^CCR4^-^ DN T cells in CTEPH patients compared to HCs (**Supplementary Figures 2A, B**). Although DN Th17 cells are less studied than the classic Th17 and Th17.1 cells, evidence was provided that these cells have pathogenic features in rheumatoid arthritis (36) and display a stable Th17-lineage commitment, even under Th1 polarization conditions (37). They have the capacity to produce IL-17, but little IFNγ and co-express the transcription factors TBX21 and RORγt, which are associated with the Th1 and Th17 subset, respectively. In this context, we confirmed intracellular expression of the key transcription factor RORγt in the total CCR6^+^ CD4^+^ T cell population (**Supplementary Figure 2C**).

Significantly more CCR6^+^ T cells were also present in the fractions of naive CD4^+^ and CD8^+^ T cells, as well as Tregs (**Figure 1**). This was not the case for CCR6^+^ memory CD8^+^ T cells, which would reflect IL-17-producing Tc17 cells (28, 38).

In conclusion, circulating T cells in CTEPH patients show an aberrant phenotype, characterized by increased proportions of CCR6^+^ cells in naive and memory CD4^+^ T cells, naive CD8^+^ T cells and Tregs.

### Increased CTLA4 Expression in CD4^+^ and CD8^+^ T Cells From CTEPH Patients

**Figure 2:**
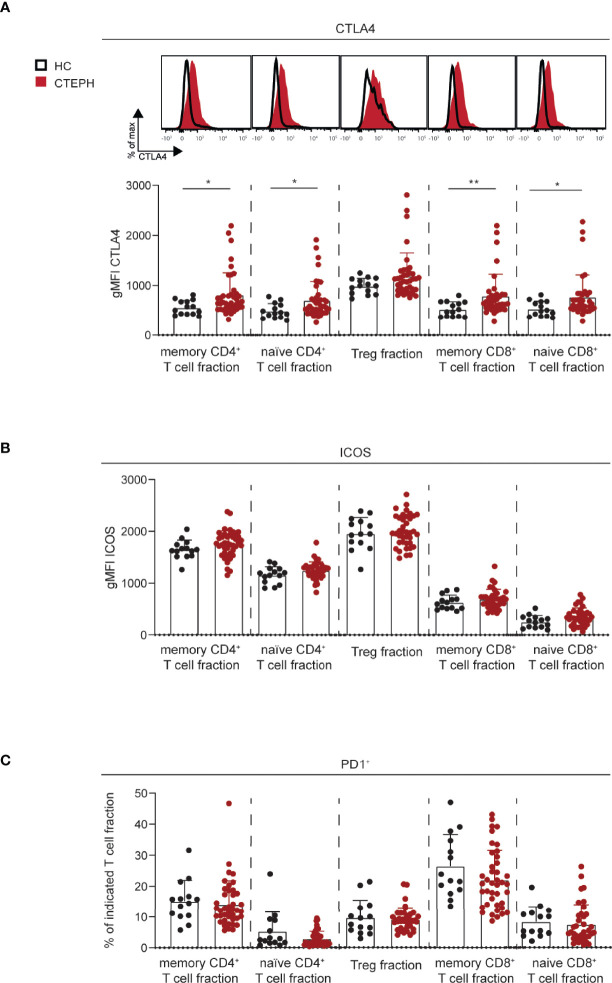
Increased CTLA4 expression in T cell subsets in CTEPH patients. **(A)** Flow cytometry analysis of intracellular CTLA4 expression in the indicated T cells subsets, shown as histogram overlays (*top*) and quantification (*bottom*). **(B)** Quantification of surface ICOS expression on the indicated T cell subsets. **(C)** Proportions of PD1^+^ cells in the indicated T cell subsets. Results are presented as mean + standard deviation, Mann-Whitney U test was used for statistical analysis, *p < 0.05, **p < 0.01. gMFI, geometric mean fluorescence intensity. Symbols represent values of individual patients or HCs.

The activation status of a T cell is reflected by the expression of activation and inhibitory markers, such as T cell co-stimulator (ICOS), programmed cell death 1 (PD1) and cytotoxic T lymphocyte antigen 4 (CTLA4) (39). Their expression was examined in the five T cell subpopulations, as gated in **Figure 1**. CTLA4 was significantly increased in all T cell fractions from CTEPH patients compared to HCs, except for Tregs in which only a slight trend was observed (**Figure 2A**). PD1 and ICOS expression were unchanged (**Figures 2B, C** and **Supplementary Figure 3A, B**). No significant correlations between the three different markers were found (shown for memory CD4^+^ T cell fractions in **Supplementary Figure 3C**).

In summary, these data indicate that both naive and memory CD4^+^ and CD8^+^ T cells of CTEPH patients have increased expression of CTLA4.

### Multivariate Analysis for T Cell Subset and Activation Marker Profile Separates Treatment-Naive CTEPH Patients From HCs

**Figure 3:**
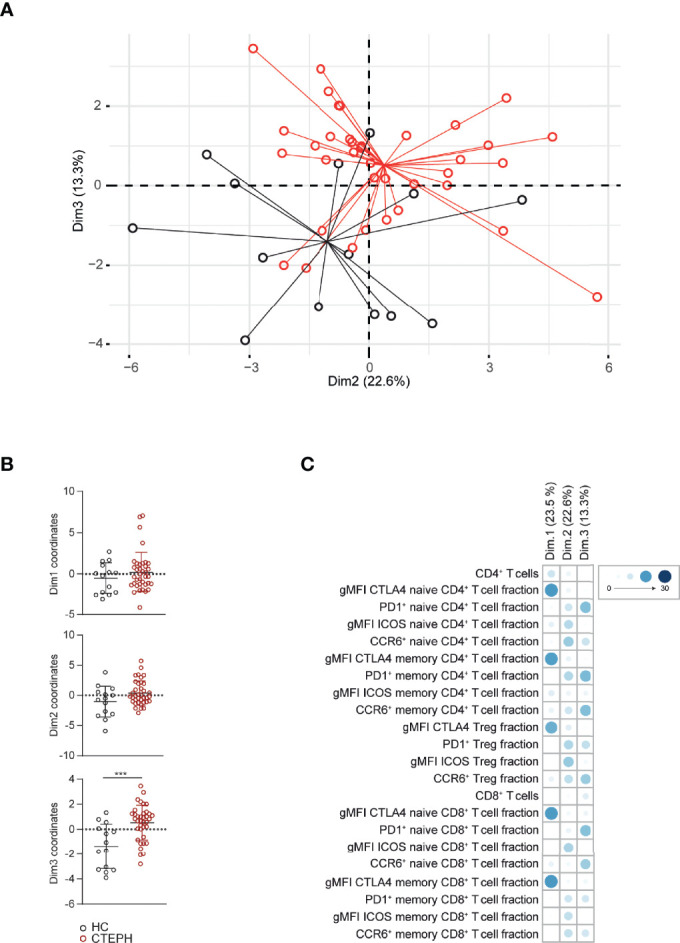
Multivariate analysis separates CTEPH patients from HCs at diagnosis. **(A)** Principal component analysis (PCA) of peripheral T cell subsets and T cell activation markers of CTEPH patients at diagnosis and HCs determined by flow cytometry **(B)** Dimension (Dim.) 1, 2 and 3 coordinate values showing the separation between patients and HCs. **(C)** Contribution of the variables in percentages indicated by symbols in blue color range (scale indicates proportions) of Dim1, Dim2 and Dim3 of the PCA. Mann-Whitney U test was used for statistical analysis of coordinates on the dimension between CTEPH patients and HCs, ***p < 0.001. Dots represent values of individual patients or HCs.

Subsequently, we used PCA to investigate if a specific T cell profile could separate treatment-naive CTEPH patients from HCs. We observed a non-random distribution that was not due to gender or age (**Figure 3A** and data not shown). The T cell profile was able to significantly separate patients from HCs in dimension 3 (Dim3) (**Figure 3B**), to which CCR6 and PD1 contributed most (**Figure 3C**). Although CTLA4 expression was significantly different between patients and HCs in most T cell populations, these differences did not significantly separate the two groups in Dim1 (**Figures 3B, C**).

Taken together, quantification of CCR6 and PD1 expression creates a T cell profile that separates CTEPH patients from HCs in a multivariate analysis.

### Reduced Cytokine-Producing Capacity of T Cells From CTEPH Patients

**Figure 4:**
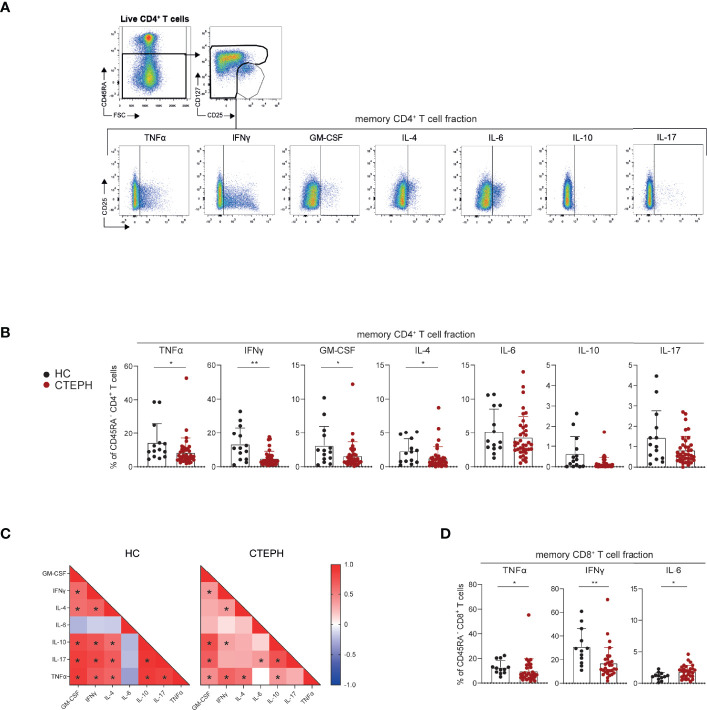
CD4^+^ and CD8^+^ T cells of CTEPH patients display reduced cytokine-producing capacity at diagnosis. **(A)** Flow cytometric gating strategy of cytokine production by circulating non-Treg CD45RA^-^ memory CD4^+^ T cells. **(B, C)** Quantification of the proportions of CD45RA^-^ CD4^+^ memory T cells expressing the indicated cytokines in HCs and CTEPH patients for CD4 memory cells **(B)** and the associated correlation matrixes for cytokine-positive cells **(C)**. **(D)** Quantification of the proportions of CD45RA^-^ CD8^+^ T cells expressing the indicated cytokines in CTEPH patients and HCs. Results are presented as mean + standard deviation, Mann-Whitney U test was used for statistical analysis, *p < 0.05, **p < 0.01. Correlation coefficient was calculated using nonparametric Spearman correlation. Dots represent values of individual patients or HCs.

Next, we determined the cytokine-producing capacity of T cells in a subgroup of 38 CTEPH patients (gating strategy as in **Figure 1**, except that Tregs were now identified by CD127^-^CD25^+^ expression; **Figure 4A**). We observed that the CD45RA^-^ memory CD4^+^ T cell fractions of CTEPH patients were less capable of producing TNFα, IFNγ, granulocyte-macrophage colony-stimulating factor (GM-CSF) and IL-4 compared with HCs, but intracellular IL-6, IL-10 and IL-17 did not differ between patients and HCs (**Figure 4B**). A correlation matrix of the proportions of cytokine-expressing CD45RA^-^CD4^+^ T cells in HCs indicated a strong coordination between all cytokines analyzed except IL-6, but this was partly lost in CTEPH patients (**Figure 4C**, examples shown in **Supplementary Figure 4**). The finding of mainly positive correlations across individual cytokines indicated that both CTEPH patients and HCs did not show skewing toward distinct Th subsets (because this would then have been visible in the matrices as negative correlations). Nevertheless, the significant positive correlation between the proportions of IL-17^+^, IFNγ^+^ and IL-4^+^ T cells in HCs was weaker in CTEPH patients. This observation suggested that in CTEPH patients the decrease of IFNγ^+^ or IL-4^+^ CD4^+^ T cells was not associated with a concomitant decrease or increase in IL-17-producing CD4^+^ T cells.

CD45RA^-^ memory CD8^+^ T cells from CTEPH patients showed reduced cytokine production, reaching significance for TNFα and IFNγ, while their IL-6 production was increased (**Figure 4D**; **Supplementary Figure 5A, B**). No differences were found in cytokine-producing capacity of CD45RA^+^ naive CD8^+^ T cells between HCs and CTEPH patients, nor in the cytokine correlation matrix (data not shown).

In summary, CD45RA^-^CD4^+^ and CD45RA^-^CD8^+^ T cells of CTEPH patients are less capable of producing cytokines, whereby correlations between the different cytokines produced by CD4^+^ T cells are weaker than in HCs.

### T Cell Cytokine Profile Separates CTEPH Patients From HCs and Separates CTEPH Patients With Central and Peripheral Lesions

Furthermore, we used PCA to investigate whether besides the T cell subset and activation marker characteristics, cytokine profiles could separate CTEPH patients from HCs (**Supplementary Figure 6A**). Indeed, cytokine T cell profiles separated patients from HCs in Dim1 to which all cytokines produced by CD4^+^ and CD8^+^ T cells contributed except IL-6 (**Supplementary Figure 6A**). A comprehensive PCA analysis that included expression of chemokine receptors, activation markers and cytokine production, showed that HCs were mainly separated from CTEPH patients by cytokine profiles (**Supplementary Figure 6B**). Given the differences in prognosis and treatment of CTEPH patients with central and peripheral lesions (5), we performed a sub-analysis comparing these two patient groups. We excluded patients with mid lesions eligible for BPA but not for PEA. We found that T cell subset and activation marker profiles did not separate patients with central (n=15) and peripheral (n=9) lesions at diagnosis (**Supplementary Figure 7A**). However, T cell cytokine profiles [determined in a smaller group of patients; central lesion (n=11) and peripheral lesion (n=6)] did separate the two patient groups in Dim2, largely based on IL-6 production by CD4^+^ and CD8^+^ T cells and GM-CSF expression by CD8^+^ T cells (**Supplementary Figures 7B, C**). We did not find significant correlations between cytokine producing abilities of CD4^+^ or CD8^+^ T cells and clinical parameters of disease course, such as patient 1-year survival (data not shown).

In conclusion, all T cell cytokines analyzed, except IL-6, constitute a T cell profile that separates CTEPH patients from HCs. In addition, CTEPH patients with central and peripheral lesions could be separated based on IL-6 and GM-CSF production by T cells.

### Changes in T Cell Phenotype in CTEPH Patients at 1-Year Follow-Up

**Figure 5:**
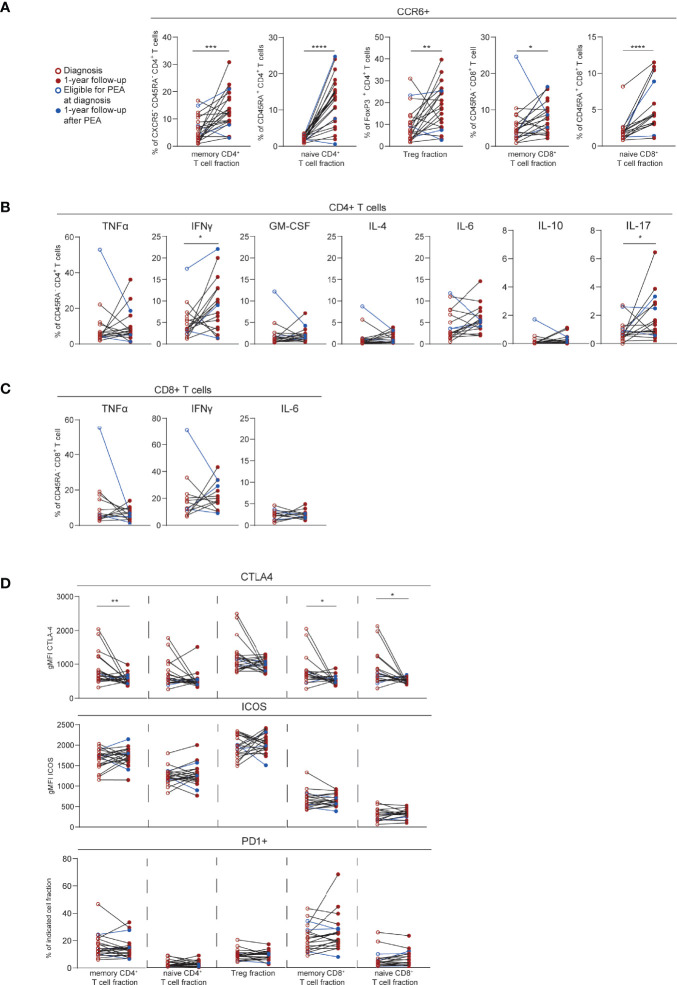
T cell phenotypic changes over time in CTEPH patients. **(A)** Quantification of circulating CCR6^+^ T cells in paired samples from CTEPH patients at diagnosis and at 1-year follow-up. **(B, C)** Proportions of cytokines-expressing cells in paired samples from CTEPH patients at diagnosis and 1-year follow up for CD45RA^-^CD4^+^ **(B)** and CD45RA^-^CD8^+^ **(C)** memory T cell subsets. **(D)** Quantification of CTLA4, PD1 and ICOS expression in the indicated T cell fractions in samples from CTEPH patients at diagnosis and 1-year follow-up. Wilcoxon matched-pairs signed rank test was used for statistical analysis, *p < 0.05, **p < 0.01, ***p < 0.001, ****p < 0.0001. gMFI = geometric mean fluorescence intensity. Closed and open circles represent values of individual patients at diagnosis or 1-year follow-up, respectively, either not eligible for PEA (*red*) or eligible for PEA (*blue*). Paired samples are connected by a line.

Next, we evaluated the T cell profiles of CTEPH patients over time. For this sub-study, we focused on a group of 22 patients of whom we had paired samples at diagnosis and at 1-year follow-up. The increase in CCR6^+^ cells in the three CD4^+^ T cell fractions analyzed (**Figure 1**) was significantly more pronounced at 1-year follow-up, especially in the CD45RA^+^CD4^+^ (naive) T cells (**Figure 5A**). An increase was also observed in the CD8^+^ T cell fractions.

Cytokine expression remained stable over time for both CD4^+^ and CD8^+^ T cell populations, except for IFNγ and IL-17 in CD4^+^ T cells, which were increased at 1-year follow-up (**Figures 5B, C**). CTLA4 expression significantly decreased over time in CD45RA^-^CD4^+^ (memory) T cells and in CD8^+^ T cells. In contrast, PD1 and ICOS expression were similar for all T cell fractions at the two time points (**Figure 5D**). Within the follow-up group of 22 patients, three patients underwent a PEA before 1-year follow-up (**Figure 5**; depicted in blue). The T cell profile of these patients at 1-year follow-up were within the range of the 19 patients who did not undergo a PEA.

These results indicate that at 1-year follow-up, CTEPH patients present with substantial changes in the expression of CCR6, cytokines and activation markers in CD4^+^ and CD8^+^ T cells, compared to baseline.

### Integration of T Cell Profile Changes of CTEPH Patients Over Time

**Figure 6:**
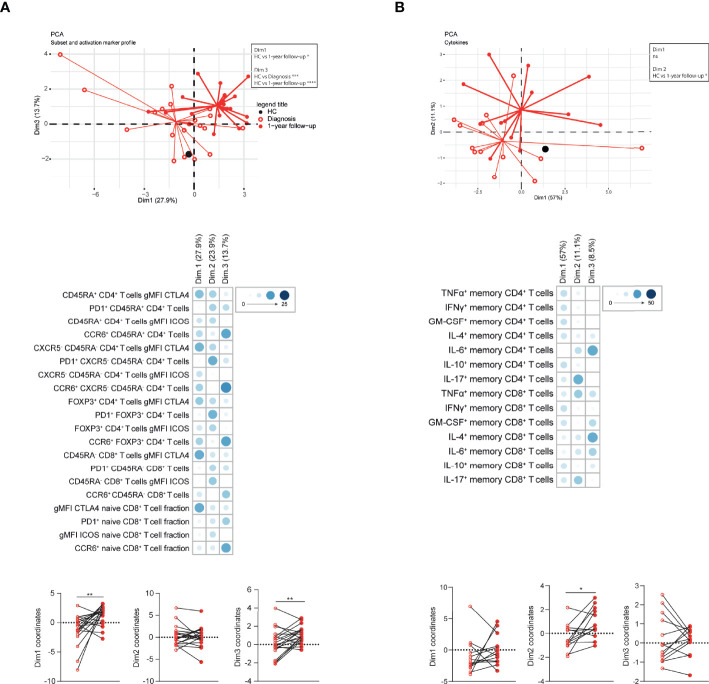
Multivariate analysis separates CTEPH patients at diagnosis and at 1-year follow-up. **(A, B)** Principal component analysis (PCA) of circulating T cell subsets and activation markers **(A)** or circulating T cell cytokine-producing capacity **(B)** of CTEPH patients and HCs by flow cytometry at diagnosis and 1-year follow-up. Lines connect the coordinates of individual patient samples to the mean coordinates of the indicated group. Mean coordinates of HCs are depicted by black dots (*top*). Contribution of the variables in percentages indicated by the blue color range for Dim1, Dim2 and Dim3 of the PCA (*middle*) and the dimension coordinate values showing the separation between samples of CTEPH patients at diagnosis (open circles) and at 1-year follow-up (closed circles)(*bottom*). Paired samples are connected by a line. Wilcoxon matched-pairs signed rank test was used for statistical analysis, **p < 0.01, *p < 0.05.

Since the T cell profile changed substantially over time, we determined whether PCA would separate CTEPH patients at diagnosis from patients at 1-year follow-up. Indeed, significant differences were observed in Dim1 and Dim3, to which CTLA4 expression and the proportions of CCR6^+^ T cells contributed most, respectively (**Figure 6A**). Likewise, patients at diagnosis and at 1-year follow-up were separated in a PCA based on T cell cytokine profiles, particularly by IL-17 in CD4^+^ and CD8^+^ T cells and TNFα in CD8^+^ T cells in Dim2 (**Figure 6B**). In these two PCAs the average positions of patient clusters over time did not move towards the position of HCs (**Figures 6A, B**).

Individual PCA analyses of patients with central and with peripheral lesions did not separate patients at diagnosis and at 1-year follow-up, using T cell subsets and activation marker profiles or cytokine expression, likely due to low sample sizes (**Supplementary Figure 8A, B**; data not shown)

Overall, integration of T cell profiles by PCA clearly separated patients at diagnosis from patients at 1-year follow-up.

### Correlation Between Phenotype of Circulating T Cells and Inflammatory Mediators in Plasma From CTEPH Patients

Next, we examined the relationship between the phenotype of circulating T cells and the concentrations of various inflammatory mediators in plasma at diagnosis determined previously (22). Various significant correlations were observed, including a positive correlation between Th17 cells (gated as CCR6^+^CXCR5^-^CD45RA^-^CD4^+^ T cells) and CXCL9 (a ligand for CXCR3) (**Supplementary Figure 9**) of which the plasma levels correlated with patient survival (22). This positive correlation would be consistent with the recent finding that CXCL9 promoted T17 differentiation in a murine model of liver disease (40). In addition, plasma levels of the anti-inflammatory cytokine IL-10 showed a negative correlation with proportions of intracellular GM-CSF^+^, IL-4^+^ and IL-17^+^ CD4^+^ T cells (**Supplementary Figure 9**), in line with the capacity of IL-10 to suppress T cell proliferative and cytokine responses (41).

Therefore, the aberrant phenotype of circulating T cells of CTEPH patients at diagnosis displayed correlations with plasma levels of cytokines and chemokines.

### Vascular Lesions of CTEPH Patients Contain CCR6^+^ CD3^+^ T Cells

**Figure 7:**
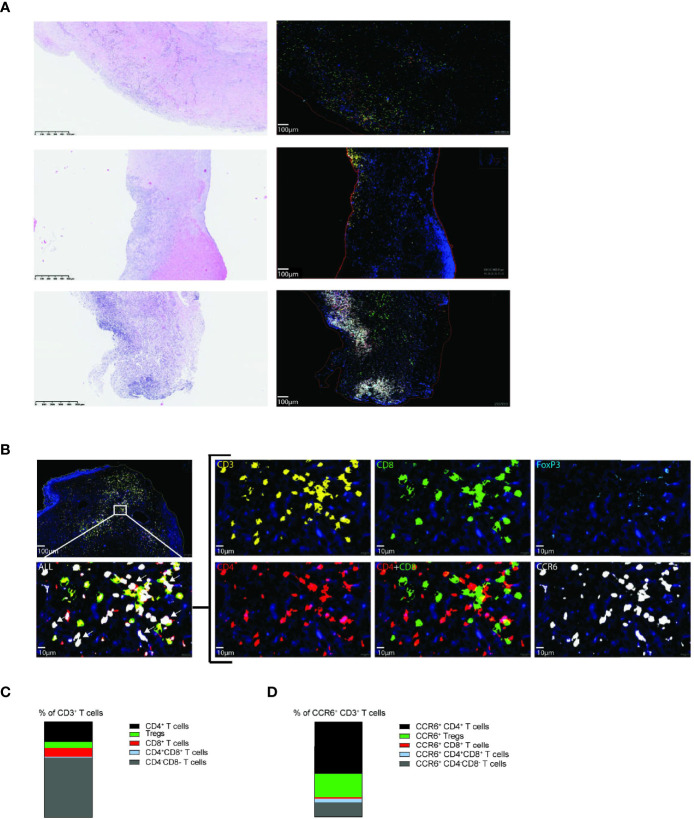
Vascular lesions of CTEPH patients contain CCR6^+^ CD3^+^ T cells. **(A)** Hematoxylin and eosin (H&E) staining (*left*) and 5-plex immunofluorescent staining of CD3 (yellow), CD4 (red), CD8 (green), FoxP3 (aqua), CCR6 (white) and Dapi (blue) (*right*) in three representative vascular lesions of CTEPH patients. **(B)** Magnification of the indicated area for immunofluorescent multiplex staining of CD4^+^ T cells (CD3+CD4+), CD8^+^ T cells (CD3+CD8+) and Tregs (CD3+CD4+FoxP3+) and their CCR6 expression. White arrows (*bottom, left*) indicate examples of CCR6^+^ CD3^+^ T cells. Scale in overview is 100 µm and in high magnifications 10 µm. **(C)** Quantification of the proportions of the indicated T cell subpopulations from total CD3^+^ T cells. **(D)** Quantification of the proportions CCR6^+^ cells within the indicated T cell subpopulations. Quantifications were by manual thresholding using Qupath in vasculature lesions of 25 CTEPH patients.

Additionally, we determined the presence of CCR6^+^ T cells in paraffin-embedded vascular lesions of 25 CTEPH patients using multiplex immunofluorescence. Vascular lesions of 19 CTEPH patients contained one or more CD3^+^ T cell clusters, the majority of which contained CCR6^+^ T cells (**Figure 7A** and **Supplementary Figure 10A**). T cell clusters were mostly located in organized thrombi and surrounding neo-vasculature, as determined by H&E staining. In the vascular lesions of the remaining 6 CTEPH patients CCR6^+^ T cells were present but were more randomly spread in the lesion (**Figure 7A**). The staining further included CD4, CD8 and FoxP3, enabling the determination of different T cell subpopulations (**Figure 7B**). Using Qupath quantification, we found that the majority of the CD3^+^ T cells were double negative (CD4^-^CD8^-^) T cells, followed by CD4^+^ T cells (**Figure 7C** and **Supplementary Figure 10B**). CCR6 expression was found on up to 60% of all T cells present, a substantial fraction of which were CD4^+^ (**Figure 7D**).

These findings demonstrate the presence of CCR6^+^CD4^+^ T cells in vascular lesions of CTEPH patients, both in T cell clusters and randomly distributed, supporting a role for CCR6^+^ T cells in pathology.

## Discussion

Although it is known that T cells are present in thrombotic lesions in the pulmonary vasculature, a detailed characterization of the T cell compartment in CTEPH patients was still lacking. We used flow cytometry to characterize circulating T cells in a well-defined treatment-naive CTEPH cohort and found that these cells have an abnormal phenotype, compared with HCs. In CTEPH patients we observed (1) that the compartments of naive CD4^+^ and CD8^+^ T cells, memory CD4^+^ T cells and Tregs contained increased proportions of CCR6^+^ cells; (2) that CD4^+^ and CD8^+^ T cells displayed reduced production of various cytokines, including TNFα and IFNγ; and (3) that in CD4^+^ and CD8^+^ T cells CTLA4 expression was increased. At 1-year follow-up, we found further increased proportions of CCR6^+^ T cells, reduced CTLA4 expression by CD8^+^ T cells and memory CD4^+^ T cells and increased IFNγ and IL-17 production by CD4^+^ T cells, compared with baseline values. Finally, we identified CCR6^+^ T cells in PEA tissue. They were found to be predominantly CD4^+^ T cells and were located in clusters as well as randomly spread over vascular lesions.

The proportions of circulating Th1 and Th2 cells did not differ between CTEPH patients and HCs. In contrast, within the memory CD4^+^ T cell population the proportions of CCR6^+^ cells were increased, pointing to enhanced T17 cell differentiation. Within the CCR6^+^ T cell fraction especially the CXCR3^-^CCR4^-^ DN Th17 cell population was increased in CTEPH patients, compared to HCs. These DN cells reflect a stable Th17-committed cell population that is likely to have pathogenic capacities in autoimmunity (36, 37). Also within the CD45RA^+^ FoxP3^-^ CD4^+^ T cell population, which mainly consists of naive CD4^+^ T cells, the proportions of CCR6^+^ cells were significantly increased in CTEPH patients. However, the CD45RA^+^ FoxP3^-^ CD4^+^ T cell population includes next to truly naive CD4^+^ T cells a small population of antigen-experienced effector memory cells re-expressing CD45RA, known as TEMRA cells. Because we observed that in healthy individuals the CCR6^+^ CD45RA^+^ FoxP3^-^ CD4^+^ T cell population contained both truly naive and TEMRA cells (DvU and IMB, unpublished results) it would be interesting to characterize this CCR6^+^CD45RA^+^ population in more detail in CTEPH patients and HCs.

Th17 cells are associated with a wide array of inflammatory conditions, including autoimmune diseases, with a strong correlation between CCR6 expression and disease severity (42). Interestingly, Th17-linked systemic autoimmune diseases including systemic sclerosis and systemic lupus erythematosus, are associated with PAH (43). Moreover, CD4^+^ T cells from idiopathic (I)PAH patients express higher levels of IL-17 and in circulating white blood cells the *IL17* gene is hypomethylated, supporting a role of Th17 cells in PAH pathology (44). Polarization of Th17 cells is promoted by IL-6 in combination with IL-1β and TGFβ, indicating an important role for IL-6 in PH. Indeed, IL-6 levels are increased in serum of IPAH patients and transgenic mouse models show that IL-6 overexpression induces PH and, conversely, IL-6 deficiency protects against hypoxia-induced PH (45–47). In plasma of CTEPH patients, IL-6 was also found to be increased (11). CCR6 expression enables Th17 cells to migrate towards inflamed tissues in response to its ligand CCL20. Accordingly, we found CCR6^+^ T cells in clusters as well as randomly spread in PEA tissue. These T cell clusters were more often located in organized thrombi than in newly formed thrombi. It is conceivable that immune cell clusters contribute to increased clotting, leading to the progression of the lesion. The T cells surrounded neo-vasculature, suggesting that part of the increased circulating CCR6^+^ T cells migrate towards the pulmonary arteries *via* this neo-vasculature into the organized thrombi. Further experiments are required to test this hypothesis. Lung CCL20 mRNA levels were found to be increased in IPAH patients, compared with controls (48) and increased CCL20 expression was associated with accumulation of CCR6^+^ and IL-17^+^ CD4^+^ T cells (45, 46). In this regard, it would be informative to determine CCL20 expression in thrombotic lesions of CTEPH patients. The striking parallels between PAH and CTEPH would support a role of Th17 cells in the etiology of both diseases, even though they are different disease entities. It remains however unknown which mechanisms contribute to the increase in CCR6^+^ Th17-lineage-associated CD4^+^ and CD8^+^ T cells in CTEPH. One of these may involve CXCL9, which has the capacity to promote Th17 differentiation (40), because we found a positive correlation between plasma levels of CXCL9 and peripheral blood Th17 cell frequencies.

Another intriguing parallel may be drawn with atherosclerosis, a chronic inflammatory arterial disease with plaque build-up in vascular lesions (49). In atherosclerosis patients, serum IL-17 and circulating Th17 cells were increased and infiltrates of IL-17-producing cells were found in atherosclerotic plaques (50). Moreover, in mouse models Th17 cells play a causative role in atherosclerosis development (51). This resemblance is striking, given the difference in embryonic origin of the pulmonary and systemic arteries affected in atherosclerosis (52).

At 1-year follow-up we observed increased production of IFNγ and IL-17 by CD4^+^ T cells and a significant further increase of the proportions of CCR6^+^ T cells. Since circulating Th1 cells were unaltered, it is attractive to speculate that the increased IFNγ production was linked to CCR6^+^ IFNγ/IL-17 double-producing Th17.1 cells (26, 38). It remains unknown if the T cell profile changes seen at 1-year follow-up were a consequence of disease progression, medication, recovery after treatment or a combination of these. Although specific PAH and CTEPH medication, such as endothelin receptor antagonists, can affect inflammatory processes (53, 54), it is unknown whether this medication affects Th17 cell differentiation or function.

Because increased CTLA4 expression can be linked to T cell exhaustion and limited DC-T cell interaction (39), it is conceivable that increased CTLA4 expression by CD4^+^ T cells contributes to the reduced cytokine-producing capacity of CD4^+^ T cells in CTEPH patients. Conversely, the decrease in CTLA4 expression at 1-year follow-up may explain the increased cytokine-producing capacity at 1-year follow-up.

Our study did not provide evidence for defects in FoxP3^+^ Tregs in CTEPH patients, except that frequencies of CCR6^+^ Tregs were increased, allowing them to migrate towards inflamed tissues. Indeed, we found CCR6^+^ Tregs in vascular lesions of CTEPH patients. The aberrant CD8^+^ T cell phenotype seen in CTEPH patients, akin to changes in CCR6, CTLA4 and cytokine expression found in CD4^+^ T cells, suggests that CD8^+^ T cells are involved in the pathogenesis of CTEPH as well. This would parallel reported findings indicating a pathogenic role of IL-17-producing CD8^+^ T cells in several auto-immune and lung diseases (29).

Our multivariate analysis based on the cytokine-producing capacity of T cells significantly separated CTEPH patients with central lesions from patients with peripheral lesions. Nevertheless, one of the limitations of our study may be that in our cohort a relatively low proportion of patients had central lesions and was therefore technically eligible for a PEA (~39%, versus ~64% in reported cohorts (2)). This makes it difficult to draw firm conclusions regarding the effects of surgery. BPA is a rather new treatment modality and few patients in our cohort underwent a BPA within 1-year follow-up. Finally, besides differences in IL-6 and GM-CSF production we did not find differences in the T cell profiles of patients with central versus peripheral lesions. This may be due to the relatively small number of patients in these subgroups.

Taken together, we found a significant increase in circulating Th17-associated CCR6^+^ T cells in CTEPH patients. Moreover, in vascular lesions CCR6^+^ T cells were found randomly spread as well as in T cell clusters. This specific CCR6^+^ profile of T cells would indicate a role of these cells in disease pathophysiology. This knowledge may help to explore avenues for the development of novel treatment strategies.

## Data Availability Statement

The raw data supporting the conclusions of this article will be made available by the authors, without undue reservation.

## Ethics Statement

The studies involving human participants were reviewed and approved by Erasmus MC medical ethical committee (MEC-2011-392). The patients/participants provided their written informed consent to participate in this study.

## Author Contributions

DvU, MK, RH, and KB conceived the project and designed the experiments. DvU, TK, JvH, TvB, MV, IB, KL, and JvT generated data or tools for the project. AvB, PB, JvT, and KB included material or patients for our study. DvU, TK, RH, and KB wrote the manuscript. All authors read, provided valuable feedback and approved the final manuscript.

## Funding

This research was partially funded by the Dutch Heart Foundation under grant number 2016T052 (DvU/MK), and partially supported by an unrestricted grant from the Pulmonary Hypertension Patients Association (TK). The authors declare this study received funding from Ferrer pharmaceuticals and Actelion Pharmaceuticals. The funders were not involved in the study design, collection, analysis, interpretation of data, the writing of this article or the decision to submit it for publication.

## Conflict of Interest

The authors declare that the research was conducted in the absence of any commercial or financial relationships that could be construed as a potential conflict of interest.

## Publisher’s Note

All claims expressed in this article are solely those of the authors and do not necessarily represent those of their affiliated organizations, or those of the publisher, the editors and the reviewers. Any product that may be evaluated in this article, or claim that may be made by its manufacturer, is not guaranteed or endorsed by the publisher.

## Abbreviations

BMI, body mass index; CTEPH, chronic thromboembolic pulmonary hypertension; 6MWT, 6-minute walk test; NT-pro BNP, The N-terminal prohormone of brain natriuretic peptide; mPAP, mean pulmonary arterial pressure; mRAP, mean right atrium pressure; PVR, pulmonary vascular resistance; PEA, pulmonary endarterectomy; BPA, Balloon pulmonary angioplasty.

## References

[1] Delcroix M Torbicki A Gopalan D Sitbon O Klok FA Lang I. ERS Statement on Chronic Thromboembolic Pulmonary Hypertension. Eur Respir J (2021) 57(6):2002828. doi: 10.1183/13993003.02828-2020 33334946

[2] Guth S D'Armini AM Delcroix M Nakayama K Fadel E Hoole SP. Current Strategies for Managing Chronic Thromboembolic Pulmonary Hypertension: Results of the Worldwide Prospective CTEPH Registry. ERJ Open Res (2021) 7(3):0085–2020. doi: 10.1183/23120541.00850-2020 PMC8365143 34409094

[3] Delcroix M Lang I Pepke-Zaba J Jansa P D'Armini AM Snijder R. Long-Term Outcome of Patients With Chronic Thromboembolic Pulmonary Hypertension: Results From an International Prospective Registry. Circulation (2016) 133(9):859–71. doi: 10.1161/CIRCULATIONAHA.115.016522 26826181

[4] Cannon JE Su L Kiely DG Page K Toshner M Swietlik E. Dynamic Risk Stratification of Patient Long-Term Outcome After Pulmonary Endarterectomy: Results From the United Kingdom National Cohort. Circulation (2016) 133(18):1761–71. doi: 10.1161/CIRCULATIONAHA.115.019470 PMC5860739 27052413

[5] Quadery SR Swift AJ Billings CG Thompson AAR Elliot CA Hurdman J. The Impact of Patient Choice on Survival in Chronic Thromboembolic Pulmonary Hypertension. Eur Respir J (2018) 52(3):1800589. doi: 10.1183/13993003.00589-2018 30002102 PMC6340636

[6] Radegran G Kjellstrom B Ekmehag B Larsen F Rundqvist B Blomquist SB. Characteristics and Survival of Adult Swedish PAH and CTEPH Patients 2000-2014. Scand Cardiovasc J (2016) 50(4):243–50. doi: 10.1080/14017431.2016.1185532 27146648

[7] Quarck R Wynants M Verbeken E Meyns B Delcroix M. Contribution of Inflammation and Impaired Angiogenesis to the Pathobiology of Chronic Thromboembolic Pulmonary Hypertension. Eur Respir J (2015) 46(2):431–43. doi: 10.1183/09031936.00009914 26113681

[8] Bochenek ML Rosinus NS Lankeit M Hobohm L Bremmer F Schutz E. From Thrombosis to Fibrosis in Chronic Thromboembolic Pulmonary Hypertension. Thromb Haemost (2017) 117(4):769–83. doi: 10.1160/TH16-10-0790 28150849

[9] Arbustini E Morbini P D'Armini AM Repetto A Minzioni G Piovella F. Plaque Composition in Plexogenic and Thromboembolic Pulmonary Hypertension: The Critical Role of Thrombotic Material in Pultaceous Core Formation. Heart (2002) 88(2):177–82. doi: 10.1136/heart.88.2.177 PMC1767204 12117850

[10] Simonneau G Torbicki A Dorfmuller P Kim N. The Pathophysiology of Chronic Thromboembolic Pulmonary Hypertension. Eur Respir Rev (2017) 26(143):160112. doi: 10.1183/16000617.0112-2016 28356405 PMC9488693

[11] Zabini D Heinemann A Foris V Nagaraj C Nierlich P Balint Z. Comprehensive Analysis of Inflammatory Markers in Chronic Thromboembolic Pulmonary Hypertension Patients. Eur Respir J (2014) 44(4):951–62. doi: 10.1183/09031936.00145013 25034560

[12] Yang M Deng C Wu D Zhong Z Lv X Huang Z. The Role of Mononuclear Cell Tissue Factor and Inflammatory Cytokines in Patients With Chronic Thromboembolic Pulmonary Hypertension. J Thromb Thrombolysis (2016) 42(1):38–45. doi: 10.1007/s11239-015-1323-2 26667361 PMC4877417

[13] Sharma S Hofbauer TM Ondracek AS Chausheva S Alimohammadi A Artner T. Neutrophil Extracellular Traps Promote Fibrous Vascular Occlusions in Chronic Thrombosis. Blood (2021) 137(8):1104–16. doi: 10.1182/blood.2020005861 33512471

[14] Aldabbous L Abdul-Salam V McKinnon T Duluc L Pepke-Zaba J Southwood M. Neutrophil Extracellular Traps Promote Angiogenesis: Evidence From Vascular Pathology in Pulmonary Hypertension. Arterioscler Thromb Vasc Biol (2016) 36(10):2078–87. doi: 10.1161/ATVBAHA.116.307634 27470511

[15] Humbert M. Pulmonary Arterial Hypertension and Chronic Thromboembolic Pulmonary Hypertension: Pathophysiology. Eur Respir Rev (2010) 19(115):59–63. doi: 10.1183/09059180.00007309 20956167 PMC9491634

[16] Koudstaal T Boomars KA Kool M. Pulmonary Arterial Hypertension and Chronic Thromboembolic Pulmonary Hypertension: An Immunological Perspective. J Clin Med (2020) 9(2):561. doi: 10.3390/jcm9020561 PMC7074374 32092864

[17] Matthews DT Hemnes AR. Current Concepts in the Pathogenesis of Chronic Thromboembolic Pulmonary Hypertension. Pulm Circ (2016) 6(2):145–54. doi: 10.1086/686011 PMC4869917 27252839

[18] Miao R Dong X Gong J Li Y Guo X Wang J. Cell Landscape Atlas for Patients With Chronic Thromboembolic Pulmonary Hypertension After Pulmonary Endarterectomy Constructed Using Single-Cell RNA Sequencing. Aging (Albany NY) (2021) 13(12):16485–99. doi: 10.18632/aging.203168 PMC8266372 34153003

[19] Tomaszewski M Grywalska E Topyla-Putowska W Blaszczak P Kurzyna M Rolinski J. High CD200 Expression on T CD4+ and T CD8+ Lymphocytes as a Non-Invasive Marker of Idiopathic Pulmonary Hypertension-Preliminary Study. J Clin Med (2021) 10(5):950. doi: 10.3390/jcm10050950 33804413 PMC7957729

[20] Deshmane SL Kremlev S Amini S Sawaya BE. Monocyte Chemoattractant Protein-1 (MCP-1): An Overview. J Interferon Cytokine Res (2009) 29(6):313–26. doi: 10.1089/jir.2008.0027 PMC2755091 19441883

[21] Yao W Firth AL Sacks RS Ogawa A Auger WR Fedullo PF. Identification of Putative Endothelial Progenitor Cells (CD34+CD133+Flk-1+) in Endarterectomized Tissue of Patients With Chronic Thromboembolic Pulmonary Hypertension. Am J Physiol Lung Cell Mol Physiol (2009) 296(6):L870–8. doi: 10.1152/ajplung.90413.2008 PMC2692803 19286928

[22] Koudstaal T van Uden D van Hulst JAC Heukels P Bergen IM Geenen LW. Plasma Markers in Pulmonary Hypertension Subgroups Correlate With Patient Survival. Respir Res (2021) 22(1):137. doi: 10.1186/s12931-021-01716-w 33947407 PMC8097895

[23] Naito A Sakao S Terada J Iwasawa S Jujo Sanada T Suda R. Nocturnal Hypoxemia and High Circulating TNF-Alpha Levels in Chronic Thromboembolic Pulmonary Hypertension. Intern Med (2020) 59(15):1819–26. doi: 10.2169/internalmedicine.4458-20 PMC7475001 32741891

[24] Langer F Schramm R Bauer M Tscholl D Kunihara T Schafers HJ. Cytokine Response to Pulmonary Thromboendarterectomy. Chest (2004) 126(1):135–41. doi: 10.1378/chest.126.1.135 15249454

[25] Olsson KM Olle S Fuge J Welte T Hoeper MM Lerch C. CXCL13 in Idiopathic Pulmonary Arterial Hypertension and Chronic Thromboembolic Pulmonary Hypertension. Respir Res (2016) 17:21. doi: 10.1186/s12931-016-0336-5 26927848 PMC4770535

[26] Stadhouders R Lubberts E Hendriks RW. A Cellular and Molecular View of T Helper 17 Cell Plasticity in Autoimmunity. J Autoimmun (2018) 87:1–15. doi: 10.1016/j.jaut.2017.12.007 29275836

[27] Tesmer LA Lundy SK Sarkar S Fox DA. Th17 Cells in Human Disease. Immunol Rev (2008) 223:87–113. doi: 10.1111/j.1600-065X.2008.00628.x 18613831 PMC3299089

[28] Kondo T Takata H Matsuki F Takiguchi M. Cutting Edge: Phenotypic Characterization and Differentiation of Human CD8+ T Cells Producing IL-17. J Immunol (2009) 182(4):1794–8. doi: 10.4049/jimmunol.0801347 19201830

[29] Srenathan U Steel K Taams LS. IL-17+ CD8+ T Cells: Differentiation, Phenotype and Role in Inflammatory Disease. Immunol Lett (2016) 178:20–6. doi: 10.1016/j.imlet.2016.05.001 PMC5046976 27173097

[30] Galie N Humbert M Vachiery JL Gibbs S Lang I Torbicki A. 2015 ESC/ERS Guidelines for the Diagnosis and Treatment of Pulmonary Hypertension: The Joint Task Force for the Diagnosis and Treatment of Pulmonary Hypertension of the European Society of Cardiology (ESC) and the European Respiratory Society (ERS): Endorsed by: Association for European Paediatric and Congenital Cardiology (AEPC), International Society for Heart and Lung Transplantation (ISHLT). Eur Respir J (2015) 46(4):903–75. doi: 10.1183/13993003.01032-2015 26318161

[31] Geenen LW Baggen VJM Koudstaal T Boomars KA Eindhoven JA Boersma E. The Prognostic Value of Various Biomarkers in Adults With Pulmonary Hypertension; A Multi-Biomarker Approach. Am Heart J (2019) 208:91–9. doi: 10.1016/j.ahj.2018.11.001 30580131

[32] Heukels P Corneth OBJ van Uden D van Hulst JAC van den Toorn LM van den Bosch AE. Loss of Immune Homeostasis in Patients With Idiopathic Pulmonary Arterial Hypertension. Thorax (2021) 76(12):1209–18. doi: 10.1136/thoraxjnl-2020-215460 PMC8606455 33963088

[33] van der Ploeg EK Golebski K van Nimwegen M Fergusson JR Heesters BA Martinez-Gonzalez I. Steroid-Resistant Human Inflammatory ILC2s Are Marked by CD45RO and Elevated in Type 2 Respiratory Diseases. Sci Immunol (2021) 6(55):eabd3489. doi: 10.1126/sciimmunol.abd3489 33514640

[34] van Uden D Koudstaal T van Hulst JAC Bergen IM Gootjes C Morrell NW. Central Role of Dendritic Cells in Pulmonary Arterial Hypertension in Human and Mice. Int J Mol Sci (2021) 22(4):1756. doi: 10.3390/ijms22041756 33578743 PMC7916474

[35] Le S Josse J Husson F. FactoMineR: An R Package for Multivariate Analysis. J Stat Software (2008) 25(1):1–18. doi: 10.18637/jss.v025.i01

[36] Dankers W den Braanker H Paulissen SMJ van Hamburg JP Davelaar N Colin EM. The Heterogeneous Human Memory CCR6+ T Helper-17 Populations Differ in T-Bet and Cytokine Expression But All Activate Synovial Fibroblasts in an IFNgamma-Independent Manner. Arthritis Res Ther (2021) 23(1):157. doi: 10.1186/s13075-021-02532-9 34082814 PMC8173960

[37] Wacleche VS Goulet JP Gosselin A Monteiro P Soudeyns H Fromentin R. New Insights Into the Heterogeneity of Th17 Subsets Contributing to HIV-1 Persistence During Antiretroviral Therapy. Retrovirology (2016) 13(1):59. doi: 10.1186/s12977-016-0293-6 27553844 PMC4995622

[38] Maggi L Santarlasci V Capone M Rossi MC Querci V Mazzoni A. Distinctive Features of Classic and Nonclassic (Th17 Derived) Human Th1 Cells. Eur J Immunol (2012) 42(12):3180–8. doi: 10.1002/eji.201242648 22965818

[39] Chen L Flies DB. Molecular Mechanisms of T Cell Co-Stimulation and Co-Inhibition. Nat Rev Immunol (2013) 13(4):227–42. doi: 10.1038/nri3405 PMC3786574 23470321

[40] Li L Xia Y Ji X Wang H Zhang Z Lu P. MIG/CXCL9 Exacerbates the Progression of Metabolic-Associated Fatty Liver Disease by Disrupting Treg/Th17 Balance. Exp Cell Res (2021) 407(2):112801. doi: 10.1016/j.yexcr.2021.112801 34461107

[41] Akdis CA Blaser K. Mechanisms of Interleukin-10-Mediated Immune Suppression. Immunology (2001) 103(2):131–6. doi: 10.1046/j.1365-2567.2001.01235.x PMC1783236 11412299

[42] Meitei HT Jadhav N Lal G. CCR6-CCL20 Axis as a Therapeutic Target for Autoimmune Diseases. Autoimmun Rev (2021) 20(7):102846. doi: 10.1016/j.autrev.2021.102846 33971346

[43] Lau EMT Giannoulatou E Celermajer DS Humbert M. Epidemiology and Treatment of Pulmonary Arterial Hypertension. Nat Rev Cardiol (2017) 14(10):603–14. doi: 10.1038/nrcardio.2017.84 28593996

[44] Hautefort A Girerd B Montani D Cohen-Kaminsky S Price L Lambrecht BN. T-Helper 17 Cell Polarization in Pulmonary Arterial Hypertension. Chest (2015) 147(6):1610–20. doi: 10.1378/chest.14-1678 25429518

[45] Soon E Holmes AM Treacy CM Doughty NJ Southgate L Machado RD. Elevated Levels of Inflammatory Cytokines Predict Survival in Idiopathic and Familial Pulmonary Arterial Hypertension. Circulation (2010) 122(9):920–7. doi: 10.1161/CIRCULATIONAHA.109.933762 20713898

[46] Steiner MK Syrkina OL Kolliputi N Mark EJ Hales CA Waxman AB. Interleukin-6 Overexpression Induces Pulmonary Hypertension. Circ Res (2009) 104(2):236–44. doi: 10.1161/CIRCRESAHA.108.182014 PMC5482545 19074475

[47] Savale L Tu L Rideau D Izziki M Maitre B Adnot S. Impact of Interleukin-6 on Hypoxia-Induced Pulmonary Hypertension and Lung Inflammation in Mice. Respir Res (2009) 10:6. doi: 10.1186/1465-9921-10-6 19173740 PMC2644669

[48] Perros F Dorfmuller P Montani D Hammad H Waelput W Girerd B. Pulmonary Lymphoid Neogenesis in Idiopathic Pulmonary Arterial Hypertension. Am J Respir Crit Care Med (2012) 185(3):311–21. doi: 10.1164/rccm.201105-0927OC 22108206

[49] Roy P Orecchioni M Ley K. How the Immune System Shapes Atherosclerosis: Roles of Innate and Adaptive Immunity. Nat Rev Immunol (2021) 22(4):251–65. doi: 10.1038/s41577-021-00584-1 PMC10111155 34389841

[50] Eid RE Rao DA Zhou J Lo SF Ranjbaran H Gallo A. Interleukin-17 and Interferon-Gamma are Produced Concomitantly by Human Coronary Artery-Infiltrating T Cells and Act Synergistically on Vascular Smooth Muscle Cells. Circulation (2009) 119(10):1424–32. doi: 10.1161/CIRCULATIONAHA.108.827618 PMC2898514 19255340

[51] Gao Q Jiang Y Ma T Zhu F Gao F Zhang P. A Critical Function of Th17 Proinflammatory Cells in the Development of Atherosclerotic Plaque in Mice. J Immunol (2010) 185(10):5820–7. doi: 10.4049/jimmunol.1000116 PMC12230985 20952673

[52] Bennett MR Sinha S Owens GK. Vascular Smooth Muscle Cells in Atherosclerosis. Circ Res (2016) 118(4):692–702. doi: 10.1161/CIRCRESAHA.115.306361 26892967 PMC4762053

[53] Montani D Humbert M Souza R. Letter by Montani Et Al Regarding Article, "Elevated Levels of Inflammatory Cytokines Predict Survival in Idiopathic and Familial Pulmonary Arterial Hypertension". Circulation (2011) 123(21):e614. doi: 10.1161/CIRCULATIONAHA.110.991596 21632514

[54] Muller DN Mervaala EM Schmidt F Park JK Dechend R Genersch E. Effect of Bosentan on NF-Kappab, Inflammation, and Tissue Factor in Angiotensin II-Induced End-Organ Damage. Hypertension (2000) 36(2):282–90. doi: 10.1161/01.HYP.36.2.282 10948091

