# Supplementary material for: Evidence for a Role of CCR6+ T Cells in Chronic Thromboembolic Pulmonary Hypertension
Source: Front Immunol. 2022 Apr 27;13:861450. doi: 10.3389/fimmu.2022.861450 (PMC9094486; doi:10.3389/fimmu.2022.861450)
Supplement: Supplementary file 1 [file DataSheet_1.pdf]

Supplementary Material

1 Supplementary Figures and Tables

1.1 Supplementary Tables

Supplementary Table 1. Monoclonal antibodies used for flow cytometry.

Antibody	Conjugate	Clone	Company
CD4	FITC	Okt4	Biolegend
CD45RA	BV650	HI100	BD
CD3	Biotin	UCHT	eBioscience
CD8	AF700	SK1	Biolegend
CD25	Pe-Cy7	M-A251	BD
CD127	BV421	A019D5	Biolegend
Streptavadin	BV605	-	BD
IL-10	PCP	JES3-9D7	Biolegend
IL-4	APC-Cy7	MP4-25D2	Biolegend
IL-6	PE	MQ2-13A5	eBioscience
IFNγ	BV711	B27	BD
IL-17a	BV786	N49-653	BD
TNFα	APC	6401.111	BD
GM-CSF	PE TxR	BVD2-21C11	BD
CCR4	FITC	-	R&D
CD45RA	PE TxR	MEM-56	Life technology
CD4	PercPcy5.5	RPA-T4	Invitrogen
CXCR5	Pe-Cy7	MU5UBEE	eBioscience
ICOS	BV650	C3984A	Biolegend
CXCR3	BV711	1C6/CXCR3	BD
PD1	BV786	EH12.1	BD
CCR6	APC	11A9	BD
CD3	APC-Cy7	UCHT1	Invitrogen
FoxP3	PE	236A/E7	Invitrogen
CTLA4	BV421	BNI3	BD

Supplementary Table 2. Antibodies used for multiplex immunofluorescence.

Antibody	Clone	Company	Cat number	Concentration
CD3	2GV6	Ventana	790-4341	0.4 ug/ml
CD4	SP35	Ventana	790-4423	2.5 ug/ml
CD8	SP57	Ventana	790-4460	0.35 ug/ml
FOXP3	236A/E7	eBioscience	14-4777-82	1/100
CCR6	polyclonal	Atlas Antibodies	HPA014488	1/50

1.2 Supplementary Figures

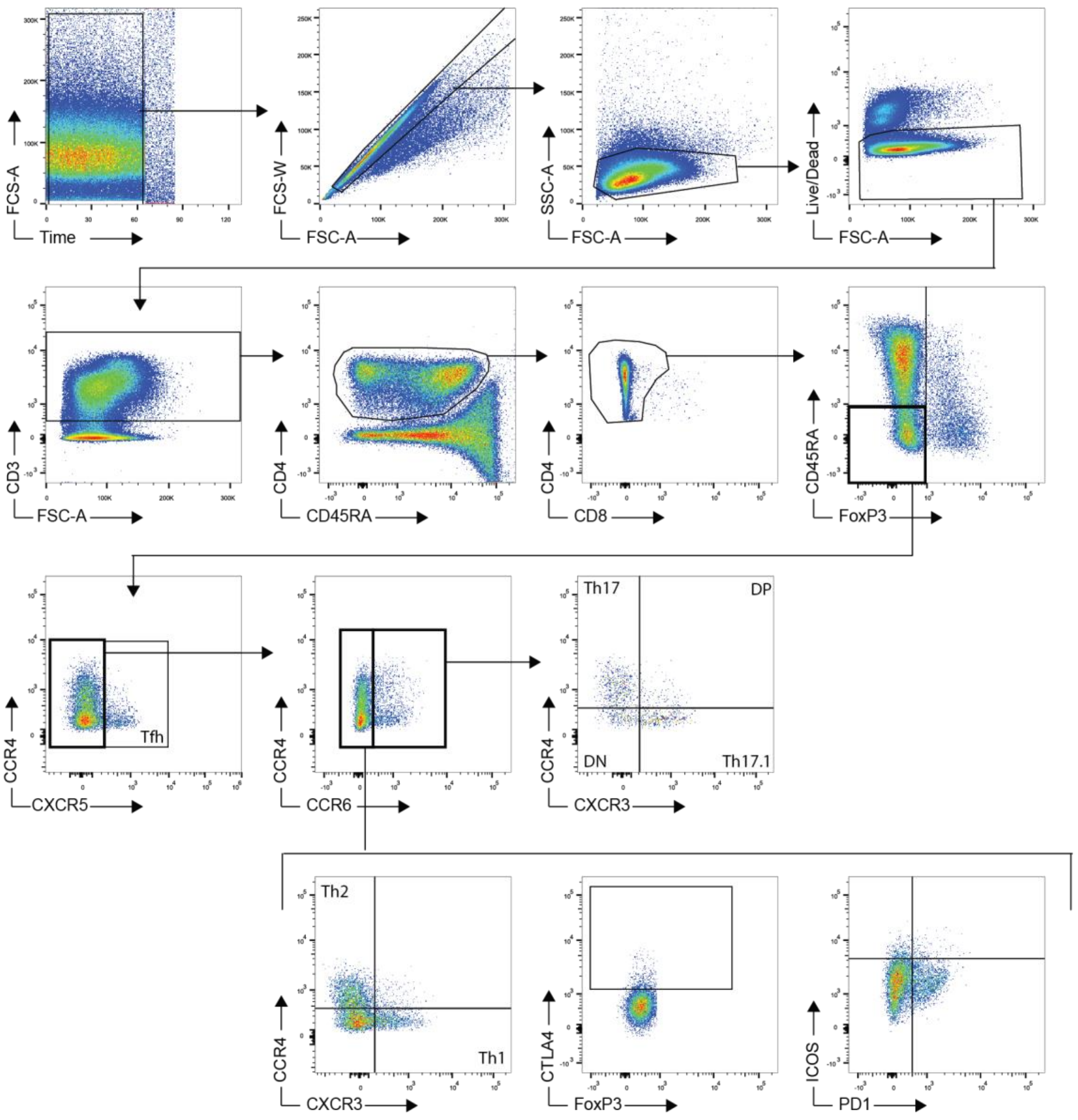

Supplementary Figure 1. Gating strategy for peripheral T cell populations. Flow cytometric gating strategy of chemokines and activation markers of peripheral blood mononuclear cells

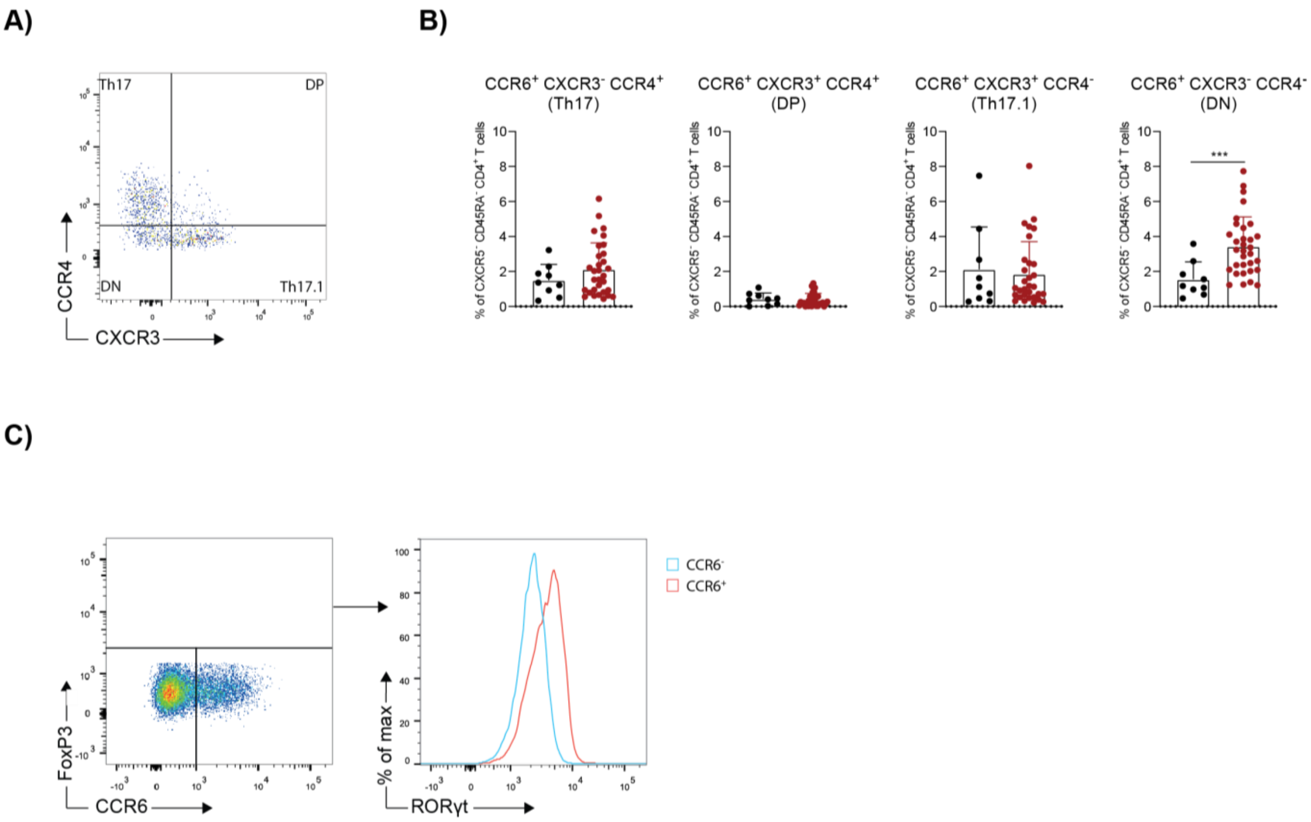

Supplementary Figure 2. Proportion of DN CCR6$^+$ T cells are significantly increased in CTEPH patients at diagnosis. (A) Flow cytometric gating strategy of CXCR3$^-$CCR4$^+$ Th17, CXCR3$^+$CCR4$^-$ Th17.1, CXCR3$^+$CCR4$^+$ and CXCR3$^-$CCR4$^-$ cells within CCR6$^+$ memory CD4$^+$ T cells. (B) Quantification of Th17, double positive (DP), Th17.1 and double negative (DN) CCR6$^+$ CD45RA$^-$ FOXP3$^-$ CD4$^+$ T cells. (C) Quantification of RORγT within CCR6$^-$ and CCR6$^+$ CD45RA$^-$ FOXP3$^-$ CD4$^+$ T cells. Results are presented as mean + standard deviation, Mann-Whitney U test was used for statistical analysis. Symbols represent values of individual patients or HCs.***= $p<0.001$.

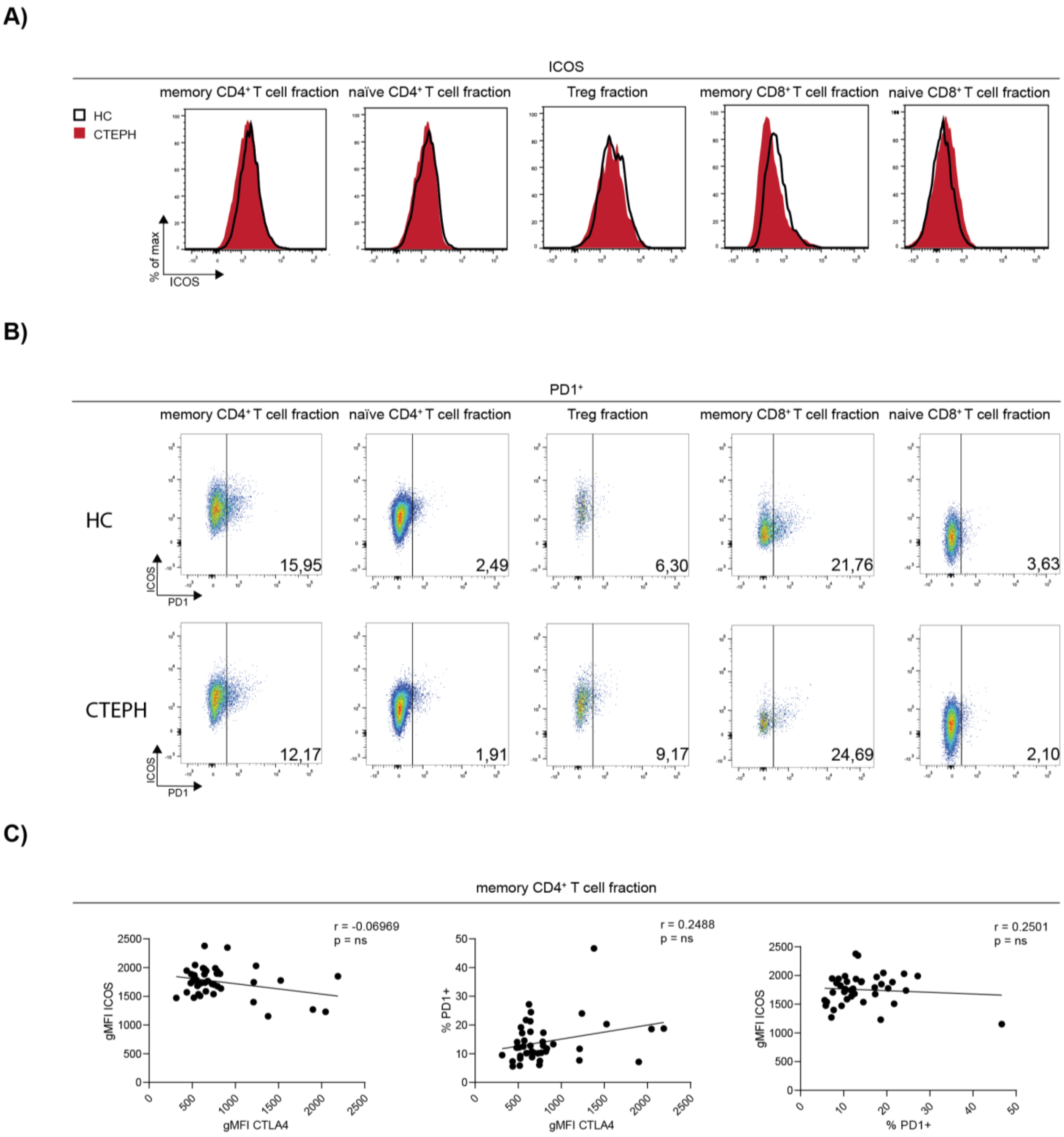

Supplementary Figure 3. Analysis of activation markers on CD4$^+$ and CD8$^+$ T cell fractions in CTEPH patients at diagnosis. (A) Flow cytometry analysis of ICOS shown as histogram overlays. (B) Flow cytometry analysis of ICOS and PD1 expression for the indicated T cell fractions, percentages of PD-1$^+$ cells is given. (C) Correlation between CTLA4, PD1 and ICOS on CXCR5$^-$ CD45RA$^-$ CD4$^+$ memory T cells. Correlation coefficients were calculated using nonparametric Spearman correlation, *p<0.05, **p<0.01, *** p< 0.001. gMFI = geometric mean fluorescence intensity. Symbols represent values of individual patients or HCs.

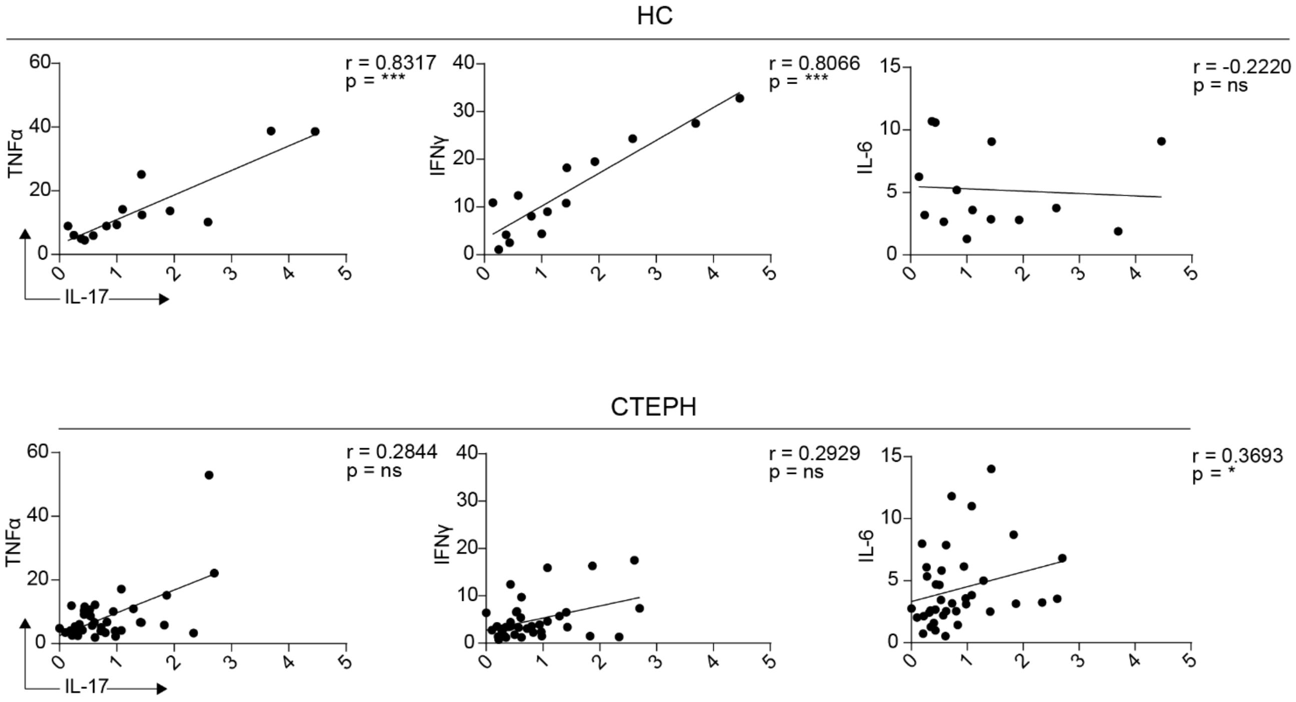

Supplementary Figure 4. IL-17 expression is correlated with TNFα and IFNγ expression in memory $CD4^+$ T cells in HCs but not in CTEPH patients at diagnosis. Correlation of $IL\text{-}17^+$ $CD45RA^-$ $CD4^+$ memory T cells with $TNF\alpha^+$, $IFN\gamma^+$ and $IL\text{-}6^+$ $CD45RA^-$ $CD4^+$ T cells in HC and CTEPH patients. Correlation coefficients were calculated using nonparametric Spearman correlation, *$p<0.05$, **$p<0.01$, *** $p< 0.001$. Symbols represent values of individual patients or HCs.

A)

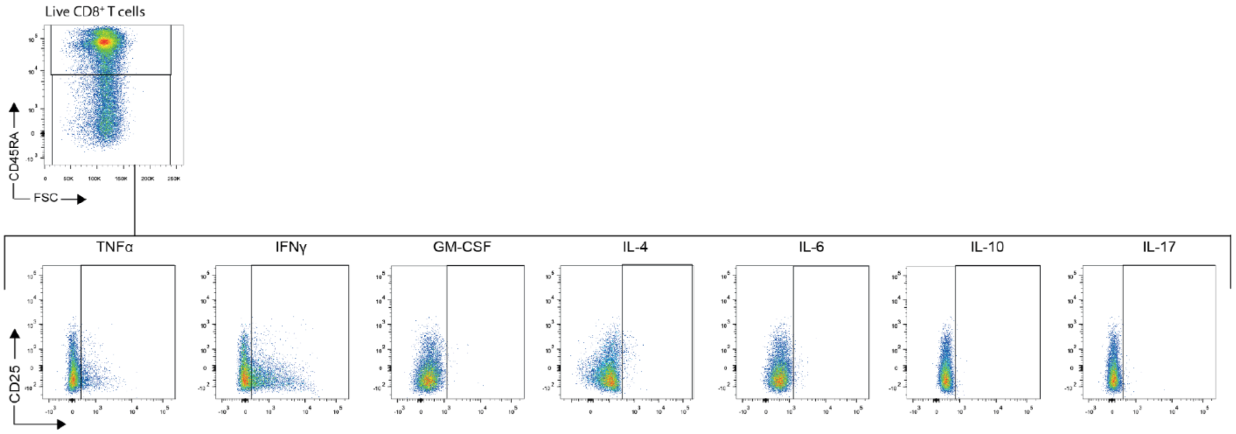

B)

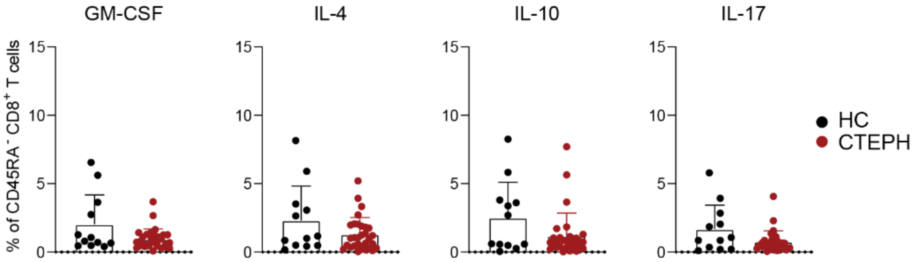

Supplementary Figure 5. Proportion of GM-CSF^{+}, IL-4^{+}, IL-10^{+} and IL-17^{+} CD45RA^{-} CD8^{+} T cells do not differ between CTEPH patients and HCs at diagnosis. (A) Flow cytometric gating strategy of cytokine production by circulating CD45RA^{-} memory CD8^{+} T cells. (B) Quantification of cytokines GM-CSF, IL-4, IL-10 and IL-17 in CD45RA^{-} CD8^{+} T cells. Results are presented as mean + standard deviation, Mann-Whitney U test was used for statistical analysis. Symbols represent values of individual patients or HCs.

A)

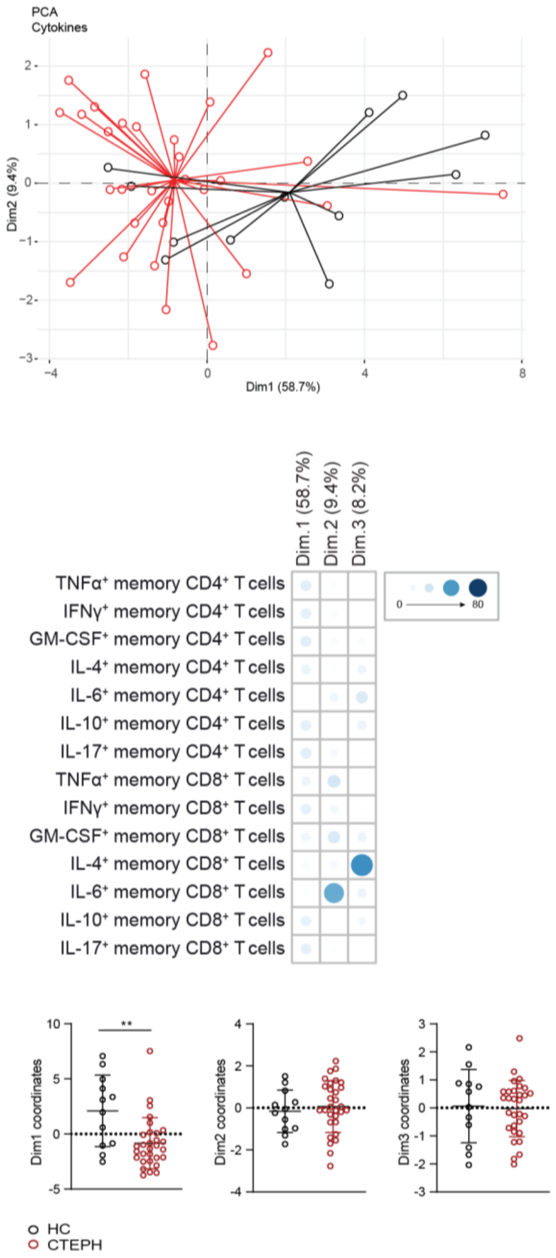
PCA
Cytokines
Dim1 (58.7%)
Dim2 (9.4%)
Dim.1 (58.7%)
Dim.2 (9.4%)
Dim.3 (8.2%)
TNFα⁺ memory CD4⁺ T cells
IFNγ⁺ memory CD4⁺ T cells
GM-CSF⁺ memory CD4⁺ T cells
IL-4⁺ memory CD4⁺ T cells
IL-6⁺ memory CD4⁺ T cells
IL-10⁺ memory CD4⁺ T cells
IL-17⁺ memory CD4⁺ T cells
TNFα⁺ memory CD8⁺ T cells
IFNγ⁺ memory CD8⁺ T cells
GM-CSF⁺ memory CD8⁺ T cells
IL-4⁺ memory CD8⁺ T cells
IL-6⁺ memory CD8⁺ T cells
IL-10⁺ memory CD8⁺ T cells
IL-17⁺ memory CD8⁺ T cells
0
80
Dim1 coordinates
Dim2 coordinates
Dim3 coordinates
**
HC
CTEPH

B)

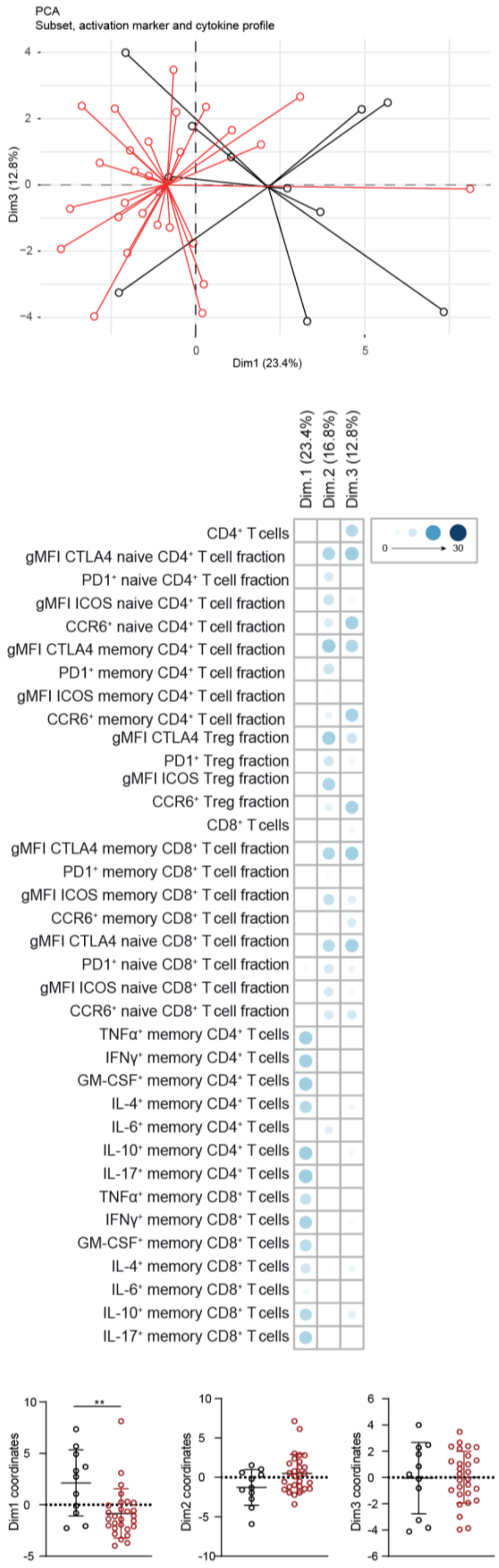
PCA
Subset, activation marker and cytokine profile
Dim1 (23.4%)
Dim3 (12.8%)
Dim.1 (23.4%)
Dim.2 (16.8%)
Dim.3 (12.8%)
CD4⁺ T cells
gMFI CTLA4 naive CD4⁺ T cell fraction
PD1⁺ naive CD4⁺ T cell fraction
gMFI ICOS naive CD4⁺ T cell fraction
CCR6⁺ naive CD4⁺ T cell fraction
gMFI CTLA4 memory CD4⁺ T cell fraction
PD1⁺ memory CD4⁺ T cell fraction
gMFI ICOS memory CD4⁺ T cell fraction
CCR6⁺ memory CD4⁺ T cell fraction
gMFI CTLA4 Treg fraction
PD1⁺ Treg fraction
gMFI ICOS Treg fraction
CCR6⁺ Treg fraction
CD8⁺ T cells
gMFI CTLA4 memory CD8⁺ T cell fraction
PD1⁺ memory CD8⁺ T cell fraction
gMFI ICOS memory CD8⁺ T cell fraction
CCR6⁺ memory CD8⁺ T cell fraction
gMFI CTLA4 naive CD8⁺ T cell fraction
PD1⁺ naive CD8⁺ T cell fraction
gMFI ICOS naive CD8⁺ T cell fraction
CCR6⁺ naive CD8⁺ T cell fraction
TNFα⁺ memory CD4⁺ T cells
IFNγ⁺ memory CD4⁺ T cells
GM-CSF⁺ memory CD4⁺ T cells
IL-4⁺ memory CD4⁺ T cells
IL-6⁺ memory CD4⁺ T cells
IL-10⁺ memory CD4⁺ T cells
IL-17⁺ memory CD4⁺ T cells
TNFα⁺ memory CD8⁺ T cells
IFNγ⁺ memory CD8⁺ T cells
GM-CSF⁺ memory CD8⁺ T cells
IL-4⁺ memory CD8⁺ T cells
IL-6⁺ memory CD8⁺ T cells
IL-10⁺ memory CD8⁺ T cells
IL-17⁺ memory CD8⁺ T cells
0
30
Dim1 coordinates
Dim2 coordinates
Dim3 coordinates
**

Supplementary Figure 6. In a multivariate analysis CTEPH patients from HCs at diagnosis are mainly separated by T cell cytokine-producing capacity. (A,B) Principal component analysis (PCA) of peripheral T cell cytokine-producing capacity (A) or of peripheral T cell subsets, T cell activation markers and cytokine production (B) of CTEPH patients and HCs determined by flow cytometry at diagnosis. Lines connect the coordinates of the patient or HC samples to their mean coordinate values (*top*). Contribution of the variables in percentages indicated by the blue color range for the indicated PCA dimensions (scale indicates proportions of the contribution) (*middle*) and Dim1, Dim2 and Dim3 coordinate values showing the separation between patients and HCs (*bottom*). Mann-Whitney U test was used for statistical analysis of coordinates on the dimension between CTEPH patients and HCs, *** $p < 0.001$. Dots represent values of individual patients or HCs.

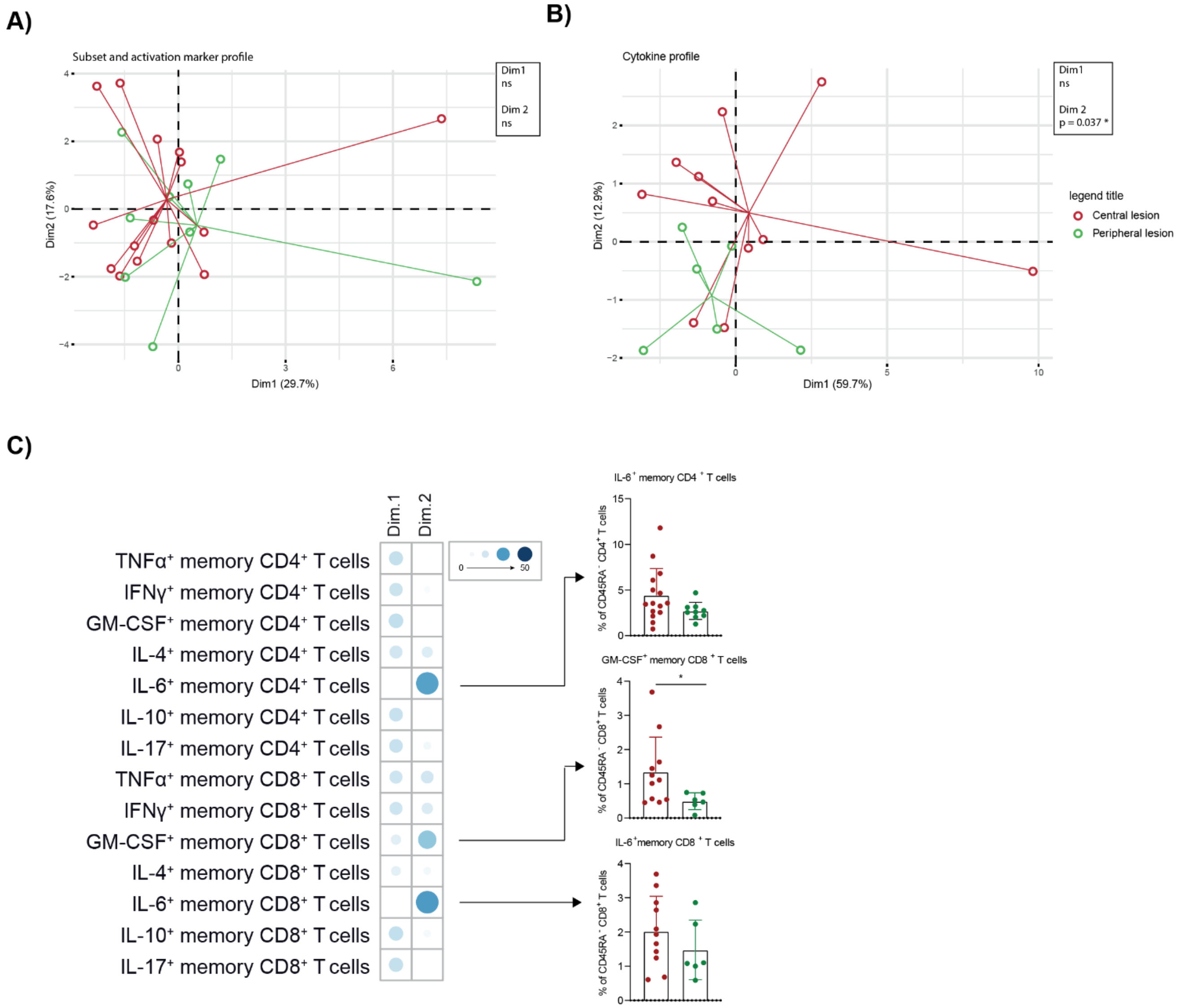

Supplementary Figure 7. IL-6 and GM-CSF production by T cells separates CTEPH patients with central lesions from CTEPH patients with peripheral lesions at diagnosis in multivariate sub-analysis. (A,B) Principal component analysis (PCA) of peripheral T cell subsets and T cell activation markers (A) or T cell cytokine-producing capacity (B) of CTEPH patients with central or peripheral lesions at diagnosis. Lines connect the coordinates of the patient samples to their mean coordinate values. (C) Contribution of the variables in percentages indicated by the blue color range for Dim1, Dim2 and Dim3 of the PCA (scale indicates proportions of the contribution). On the right-side flow cytometry analysis of IL-6^{+} CD4^{+} and CD8^{+} T cells and GM-CSF^{+} CD8^{+} T cells. Results are presented as mean + standard deviation, Mann-Whitney U test was used for statistical analysis between CTEPH patients and HCs, **$p<0.01$, *** $p< 0.001$. Red circles represent individual CTEPH patients with central lesions and green circles represent CTEPH patients with peripheral lesions.

A) B)

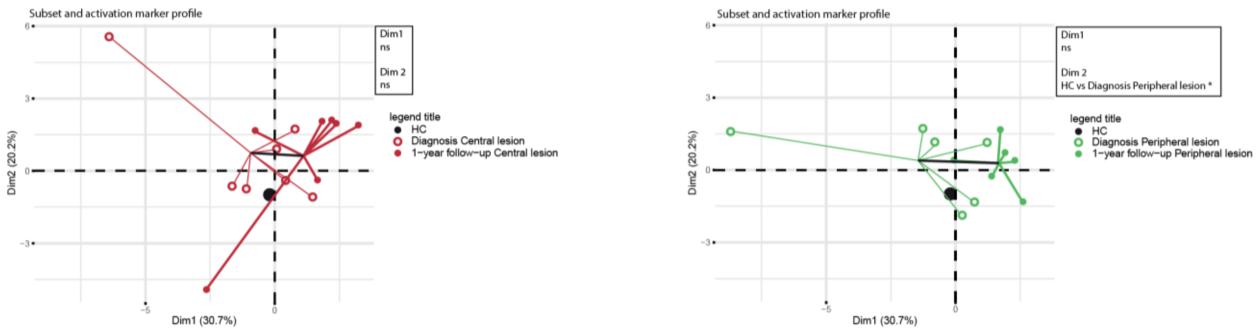

Supplementary Figure 8. PCA does not distinguish CTEPH patients at diagnosis from patients at 1-year follow-up in a separate analysis of patients with central and peripheral lesions. (A,B) Principal component analysis (PCA) of peripheral T cell subsets and T cell activation markers of CTEPH patients with central (A) or peripheral lesions (B) at diagnosis or 1-year follow-up and HCs determined by flow cytometry. Lines connect the coordinates of the patient samples to their mean coordinate values. Mann-Whitney U test was used for statistical analysis of coordinates on Dim1 and Dim2 between CTEPH patients and HCs, **$p<0.01$, *** $p< 0.001$. Open circles represent individual CTEPH patients at diagnosis and closed circles represent individual CTEPH patients at 1-year follow-up. Black symbols indicate mean coordinates of HCs.

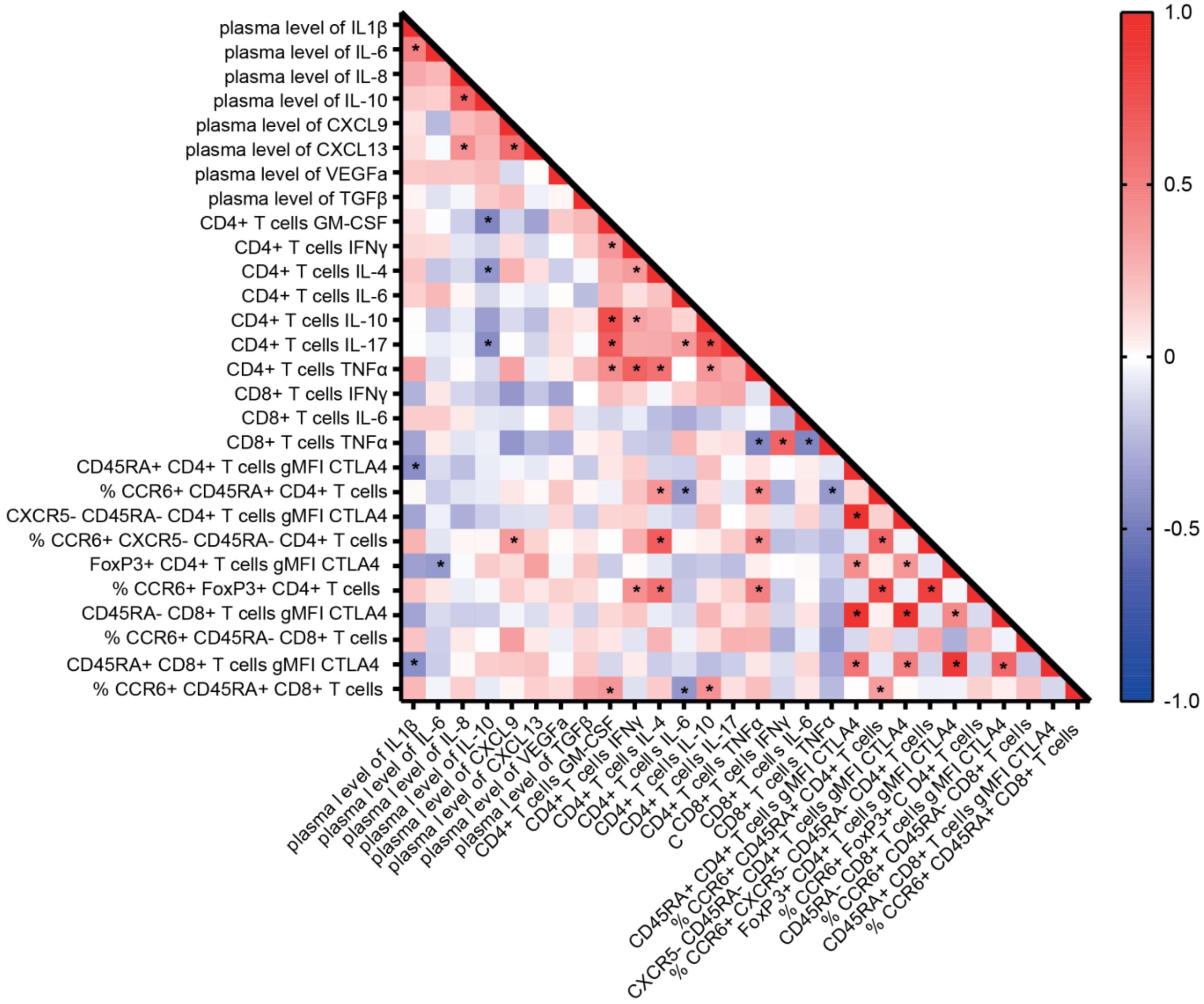

Supplementary Figure 9. Correlation of phenotype of circulating T cells and plasma levels of inflammatory mediators in CTEPH patients. Correlation Matrix of plasma levels of the indicated cytokines, chemokines and vascular growth factors and flow cytometry data of CD4^{+} and CD8^{+} T cells of CTEPH patients. Scale indicates correlation coefficient. Correlation coefficient was calculated using nonparametric Spearman correlation, *p<0.05.

A)

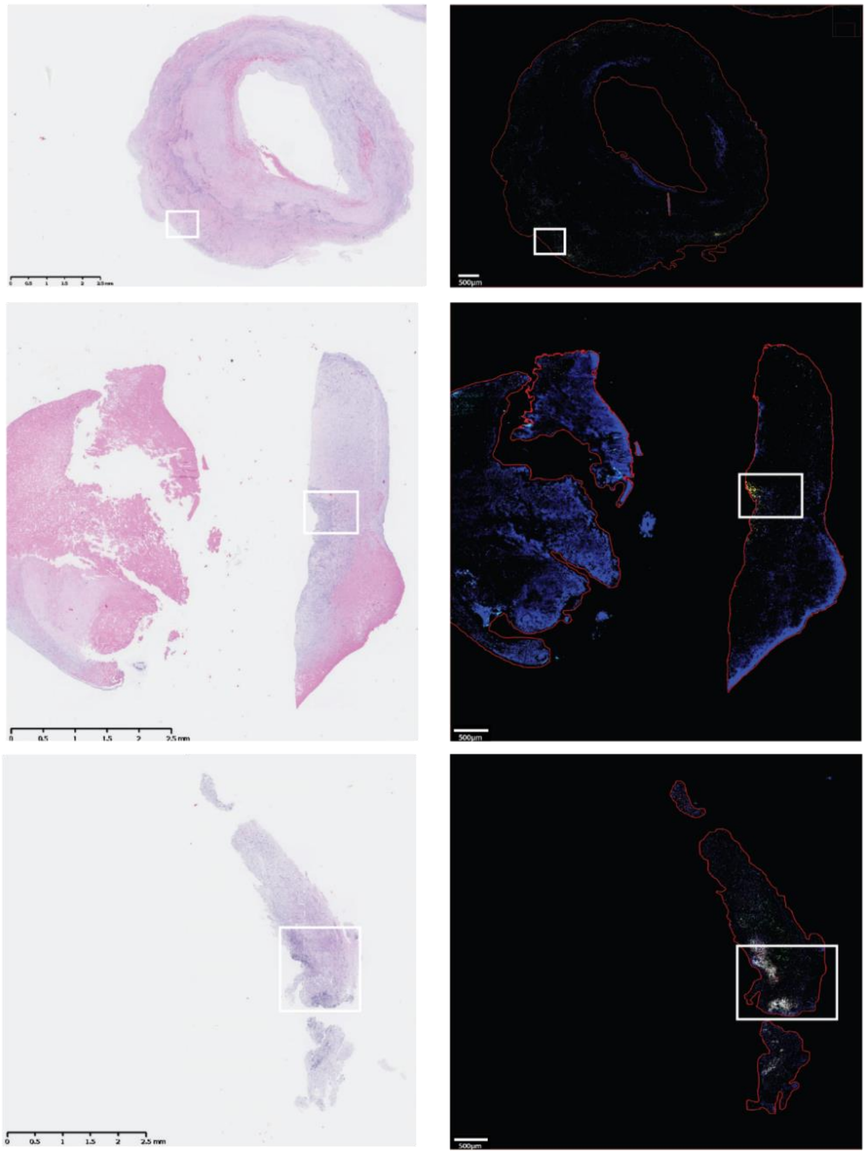
0 0.5 1 1.5 2 2.5 mm
500μm
0 0.5 1 1.5 2 2.5 mm
500μm
0 0.5 1 1.5 2 2.5 mm
500μm

B)

1.

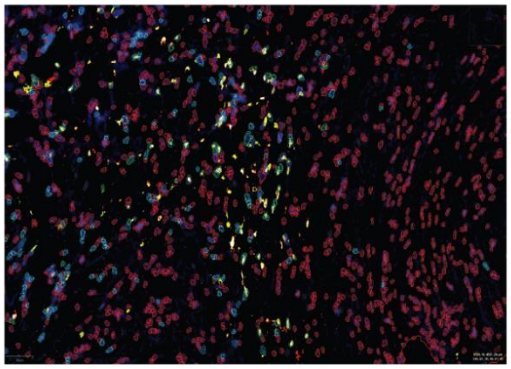

2.

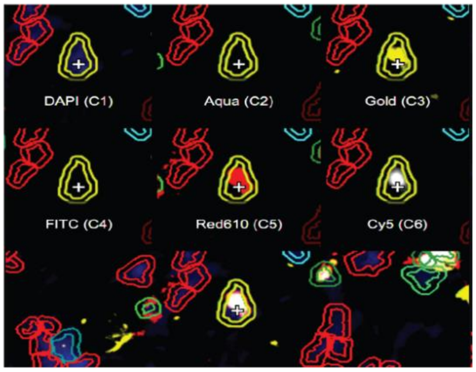
DAPI (C1)
Aqua (C2)
Gold (C3)
FITC (C4)
Red610 (C5)
Cy5 (C6)

Supplementary Figure 10. Immunofluorescence multiplex staining to determine CCR6$^+$ T cells in vascular lesions of CTEPH patients. (A) Overview of hematoxylin and eosin (H&E) staining (*left*) and 5-multiplex staining (*right*) of selected areas for main Figure 8. (B) Example illustrating the method used to identify cells (*panel 1, left*) and determine marker positivity by manual thresholding (*panel 2, right*) in Qupath.
